# Role of the inflammasome in insulin resistance and type 2 diabetes mellitus

**DOI:** 10.3389/fimmu.2023.1052756

**Published:** 2023-03-13

**Authors:** Shen Lu, Yanrong Li, Zhaojun Qian, Tiesuo Zhao, Zhiwei Feng, Xiaogang Weng, Lili Yu

**Affiliations:** ^1^ The Third Affiliated Hospital of Xinxiang Medical University, Xinxiang, Henan, China; ^2^ School of Basic Medical Sciences, Xinxiang Medical University, Xinxiang, Henan, China; ^3^ Institute of Precision Medicine, Xinxiang Medical University, Xinxiang, Henan, China; ^4^ Xinxiang Key Laboratory of Tumor Vaccine and Immunotherapy, Xinxiang Medical University, Xinxiang, Henan, China

**Keywords:** inflammasome, insulin resistance, type 2 diabetes mellitus, therapeutic agents, NLRP3

## Abstract

The inflammasome is a protein complex composed of a variety of proteins in cells and which participates in the innate immune response of the body. It can be activated by upstream signal regulation and plays an important role in pyroptosis, apoptosis, inflammation, tumor regulation, etc. In recent years, the number of metabolic syndrome patients with insulin resistance (IR) has increased year by year, and the inflammasome is closely related to the occurrence and development of metabolic diseases. The inflammasome can directly or indirectly affect conduction of the insulin signaling pathway, involvement the occurrence of IR and type 2 diabetes mellitus (T2DM). Moreover, various therapeutic agents also work through the inflammasome to treat with diabetes. This review focuses on the role of inflammasome on IR and T2DM, pointing out the association and utility value. Briefly, we have discussed the main inflammasomes, including NLRP1, NLRP3, NLRC4, NLRP6 and AIM2, as well as their structure, activation and regulation in IR were described in detail. Finally, we discussed the current therapeutic options-associated with inflammasome for the treatment of T2DM. Specially, the NLRP3-related therapeutic agents and options are widely developed. In summary, this article reviews the role of and research progress on the inflammasome in IR and T2DM.

## 1 Introduction

According to the latest global diabetes map (10th edition) released by the International Diabetes Federation (IFD), the number of adult diabetics worldwide is increasing at an alarming rate, 74 million more than in 2019, an increase of 16%. By 2021, about 537 million adults (aged 20-79) worldwide had diabetes, accounting for 10.5% of the global adult population. It is estimated that the total number of diabetic patients in the world will increase to 643 million (11.3%) and 783 million (12.2%) by 2030 and 2045, respectively. At present, the number of diabetic patients in China ranks first in the world, about 140 million, and half of these patients are unaware that they have diabetes (1). In the diabetic population, T2DM accounts for more than 90% of all diabetic cases. IR is one of the main pathophysiological characteristics of T2DM, and widely exists in obesity, nonalcoholic fatty liver disease (NAFLD), hypertension, hyperlipidemia, polycystic ovary syndrome, cardiac metabolic syndrome and other metabolic diseases (2).

**Figure 1:**
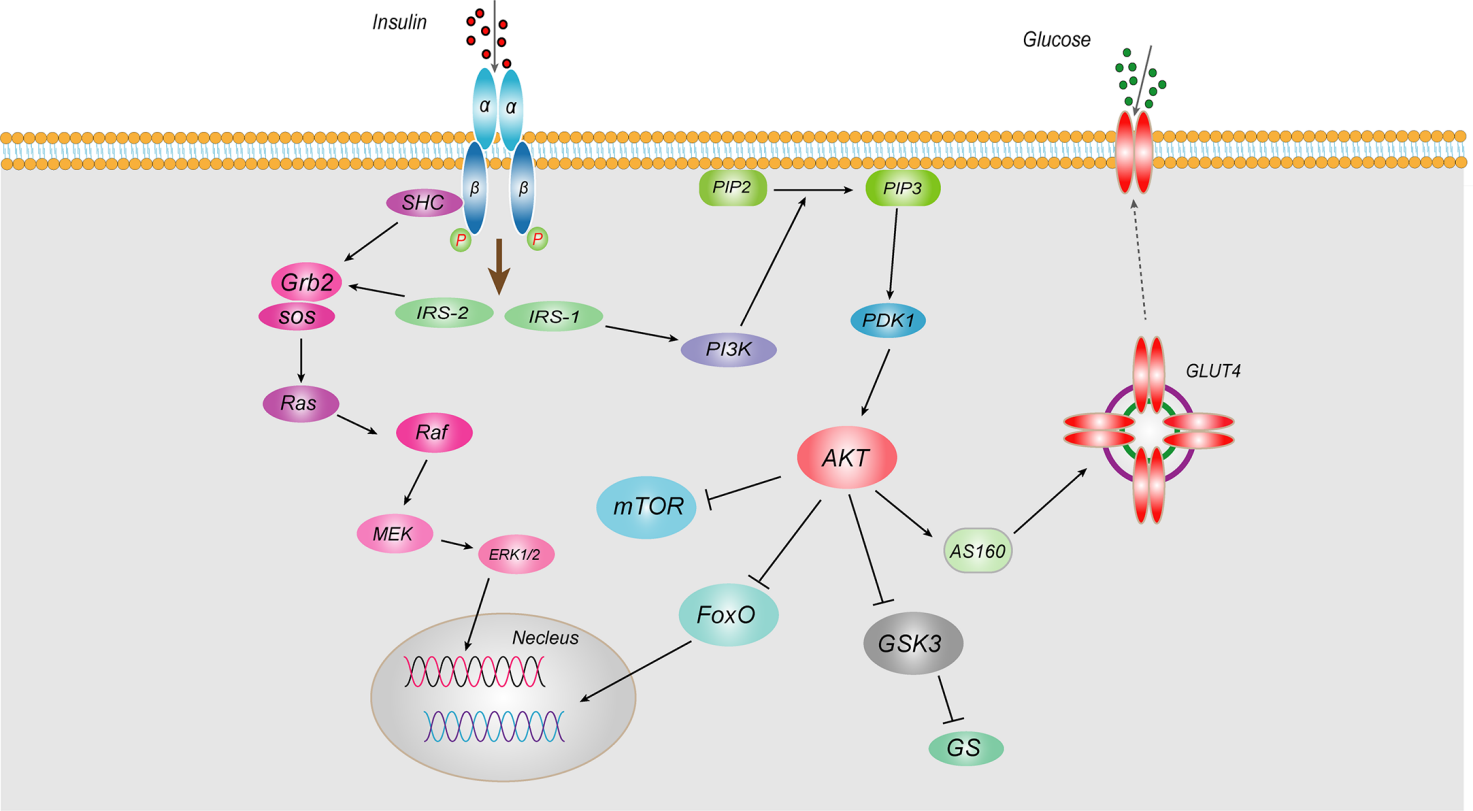
Schematic representation of insulin signaling pathway Insulin works mainly through two pathways, the PI3K and MAPKs. As for PI3K signaling pathway, insulin binds to the α-subunit of the INSR on the cell membrane of target tissues, leading to phosphorylation of the β subunit tyrosine site in the cytoplasm. Then activated IRS-1 binds to the regulatory p85 subunit of downstream PI3K, thereby leading to the phosphorylation of Akt, which in turn phosphorylates AS160 and promotes the transport of GLUT4 from intracellular vesicles to the plasma membrane, increasing transport of glucose into the cell and promoting synthesis of hepatic glycogen, inhibiting glycogen decomposition, and reducing blood glucose concentrations. Akt also can inhibit the activity of nuclear transcription factor FoxO, thereby inhibiting gluconeogenesis. Regarding another MAPK signaling pathway, after insulin binding with INSR, the activated IRS-2 can bind to the SH2 segment of Grb2, and then acts on the signaling protein GDP/GTP exchange factor to convert the inactive Ras-GDP into active Ras-GTP. Activated Ras recruits Raf serine kinase, which can phosphorylates MAPKK serine, a MAPK kinase that activates MAPK. Activated MAPK can induce phosphorylation of various target proteins that participate in the phosphorylation of ERK1/2 to regulate cell proliferation, cell variation, angiogenesis, DNA repair, etc.

IR, known as “decreased insulin sensitivity”, refers to the lower biological effects of normal doses of insulin on target tissues in healthy people. Insulin mainly acts on peripheral tissues (muscle, liver and adipose tissue), promoting the uptake and utilization of glucose and glycogen synthesis and inhibiting hepatic gluconeogenesis. IR is mainly caused by abnormal insulin signaling pathways, referring to a series of abnormalities in signaling transmission to cells after insulin binds to insulin receptor (INSR). Insulin works mainly through two pathways, the phosphatidylinositol 3-kinase (PI3K) and mitogen-activated protein kinase (MAPK) (3) (**Figure 1**). As for PI3K signaling pathway, insulin binds to the α-subunit of the INSR on the cell membrane of target tissues, leading to phosphorylation of the β subunit tyrosine (Tyr) site in the cytoplasm. Then activated insulin receptor substrates (IRS-1 and IRS-2) binds to the regulatory P85 subunit of downstream PI3K, thereby leading to the phosphorylation of Akt, which in turn phosphorylates Akt substrate 160 (AS160) and promotes the transport of glucose transporter 4 (GLUT4) from intracellular vesicles to the plasma membrane, increasing transport of glucose into the cell and promoting synthesis of hepatic glycogen, inhibiting glycogen decomposition, and reducing blood glucose concentrations (4). Akt also can phosphorylate and inhibits the activity of nuclear transcription factor Forkhead box transcription factor O (FoxO), thereby inhibiting gluconeogenesis (5). Regarding another MAPK signaling pathway, after insulin binding with INSR, the activated IRS-2 can bind to the SH2 segment of growth factor receptor binding protein 2 (Grb2), and then acts on the signaling protein guanosine diphosphate (GDP)/guanosine triphosphate (GTP) exchange factor to convert the inactive Ras-GDP into active Ras-GTP. Activated Ras recruits Raf serine kinase, which can phosphorylates MAPKK serine, a MAPK kinase that activates MAPK. Activated MAPK can induce phosphorylation of various target proteins that participate in the phosphorylation of extracellular regulated protein kinase 1/2 (ERK1/2) to regulate cell proliferation, cell variation, angiogenesis, DNA repair, etc. Any abnormality in the insulin signaling pathway leads to obstruction of the insulin signaling pathway and induces IR. Epidemiological studies have shown that IR is present in more than 80% of patients with T2DM. Therefore, it is of great significance to explore the pathogenesis and related mechanisms of IR.

There is a clear relationship between the chronic activation of pro-inflammatory signaling pathways and IR, such as c-Jun N-terminal kinase (JNK) and Nuclear Factor Kappa-B (NF-κB) (4, 6). In addition, inflammatory cytokines are also closely related to abnormal insulin signaling pathways. Tumor necrosis factor-α (TNF-α), interleukin-1 (IL-1), monocyte chemoattractant protein-1 (MCP-1), and C-reactive protein can lead to an intracellular inflammatory response and activate the signaling of inflammatory cytokines, blocking intracellular insulin signaling in target tissues and triggering IR (7, 8). Recently, increased studies have shown that inflammasomes are multi-protein complexes assembled by NOD-like receptors (NLRs) and AIM2-like receptors (ALRs) in the cytoplasm, playing the core role in regulating inflammatory response. The activation and signal transduction of the inflammasome are closely related to the occurrence and development of various metabolic diseases. Moreover, inflammasomes can directly or indirectly affect conduction of the insulin signaling pathway, involvement the occurrence of IR and T2DM. Until now, there is no comprehensive description of the relationship between a variety of common inflammasomes and IR. Thus, this review will discuss the roles of common inflammasomes in IR and T2DM. Particularly, the different kinds of therapeutic agents through regulation of inflammasomes for T2DM treatment were summarized.

## 2 Inflammasomes

**Figure 2:**
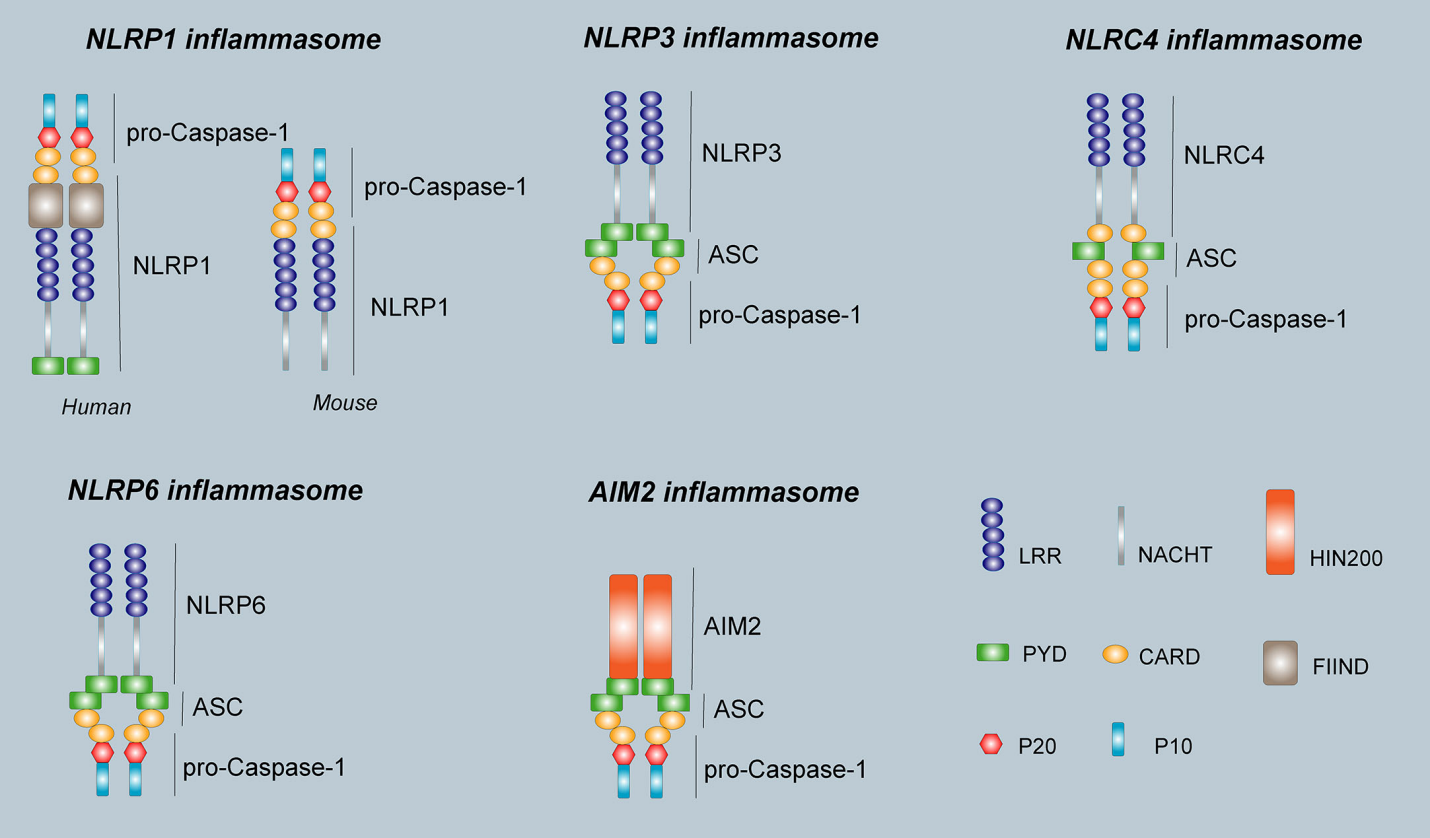
The structure of inflammasomes. The human NLRP1 receptor protein consists mainly of a PYD domain, a NACHT domain, a C- terminal LRR domain, a FIIND domain, and a CARD domain, however, the mouse NLRP1b receptor protein does not have PYD domain and FIIND domain. The NLRP3 receptor protein is the same as NLRP6, and consists of an N-terminal effector domain PYD, an intermediate-terminal NACHT domain, and a C-terminal LRR. The NLRC4 receptor protein consists of an N-terminal effector domain CARD, an intermediate-terminal NACHT domain, and a C-terminal LRR. The AIM2 receptor protein consists of two parts, the N-terminal PYD and a HIN domain at the C-terminus. The ASC consists of PYD and CARD. pro-Caspase-1 is composed of P20 and P10. The inflammasome consists of a receptor protein, ASC, and pro-Caspase-1.

In 2002, Martinon et al. (9) first proposed the concept of the inflammasome. The inflammasome is a complex composed of a variety of proteins with a relative molecular mass of 700 kDa that plays an important role in resisting external pathogen infection and abnormal self-generated hazard signals. The basic structure of the inflammasome includes the receptor proteins, such as NLRP1, NLRP3, NLRC4, NLRP6, NLRP7, and NLRP12 in the NLRs family or AIM2 and IFI16 in the ALRs family. The apoptosis-associated speck-like protein containing a CARD (ASC) are used as linker proteins and Caspase-1, Caspase-4, Caspase-5, Caspase-11, within the Caspase family, are used as effector proteins. Some inflammasomes formed by NLRP1, NLRP3, NLRC4, NLRP6, and AIM2 receptor proteins are called canonical inflammasomes and can promote the activation of Caspase-1 and produce inflammatory reactions. Other noncanonical inflammasomes can trigger a series of immune responses by promoting the activation of Caspase-4, Caspase-5, and Caspase-11 (10). Different types of inflammasomes have different compositions and functions (**Figure 2**).

The inflammasome activation pathway is generally divided into two steps. The first step is activation of inflammasome-related precursors. For example, the NF-κB signaling pathway is activated when stimuli are upregulated. NF-κB translocated to the nucleus, leading to activation of receptor molecules, pro-IL-1β and pro-IL-18. The second step in inflammasome activation includes a variety of exogenous and endogenous stimuli. The pyrin domain (PYD) and Caspase activation and recruitment domain (CARD) of the inflammasome can oligomerize activated sensor proteins and recruit an ASC adapter. Aggregation of ASCs into micron-sized ASC spots through CARD-CARD interaction, and then pro-Caspase-1/11 is recruited (10). Activated Caspase-1/11 can cleave pro-IL-1β and pro-IL-18 in the medial aspect of the cytoplasm, and finally the mature IL-1β and IL-18 are released from the cell, promoting the inflammatory reaction.

## 3 Role of the Inflammasome in insulin resistance

### 3.1 NLRP1 inflammasome

#### 3.1.1 Structure and activation of NLRP1 inflammasome

The NLRP1 inflammasome was the first discovered protein complex known to activate Caspase-1 and IL-1β. NLRP1 is located in the human coding gene 17p13 and widely exists in a variety of immune cells, the glandular epithelium of the gastrointestinal tract and respiratory tract, neurons, and non-hematopoietic cells (11). There is only one gene encoding the human NLRP1 protein, whereas three different gene encode NLRP1a, NLRP1b, and NLRP1c in mice (12). NLRP1 is mainly composed of PYD, a nucleotide-binding and oligomerization (NACHT) domain, a C-terminal leucine-rich repeat (LRR), a function-to-find domain (FIIND) and a CARD domain (13) **(** **Figure 2** **)**. PYD and LRR play vital roles in maintaining the functions of the active monomers of the NLRP1 inflammasome. The CARD domain at the C-terminus of NLRP1 can directly bind to pro-Caspase-1 without the need of the linker protein ASC. However, ASC participation can significantly increase the activity of NLRP1 inflammasome (14).

Three kinds of NLRP1 ligands have been identified, namely, *bacillus anthracis* lethal toxin (BALT) (15), muramyl dipeptide (MDP) (16), and *Toxoplasma gondii* (17). Additionally, the ligands of NLRP1 are species-specific. For example, BALT can only activate NLRP1 in mice, whereas MDP can only activate NLRP1 in humans. The specific mechanism of NLRP1 activation is not completely clear. It has been reported that under the stimulation of BALT, MAPKK in the cytoplasm is cleaved and activated, which leads to K^+^ efflux from the cytoplasm, instability of cells, and hydrolysis-induced activation of NLRP1 inflammasome in the host cell (18). Ca^2+^ efflux and proteasome activation also play important roles in the activation of NLRP1 inflammasome (19, 20). In addition, the latest study found two physiological stimulators of human NLRP1. One is that Robinson et al. (21) reported that NLRP1 could be cleaved by 3C protease at its N-terminus. After cleavage, the N-terminal fragment was degraded, and UPA-CARD at the C-terminus was activated to form inflammasomes. Another is that Bauernfred et al. (22) found that double-stranded RNA can also activate NLRP1 when infection with *Semliki Forest Virus.*

#### 3.1.2 NLRP1 inflammasome and IR

**Figure 3:**
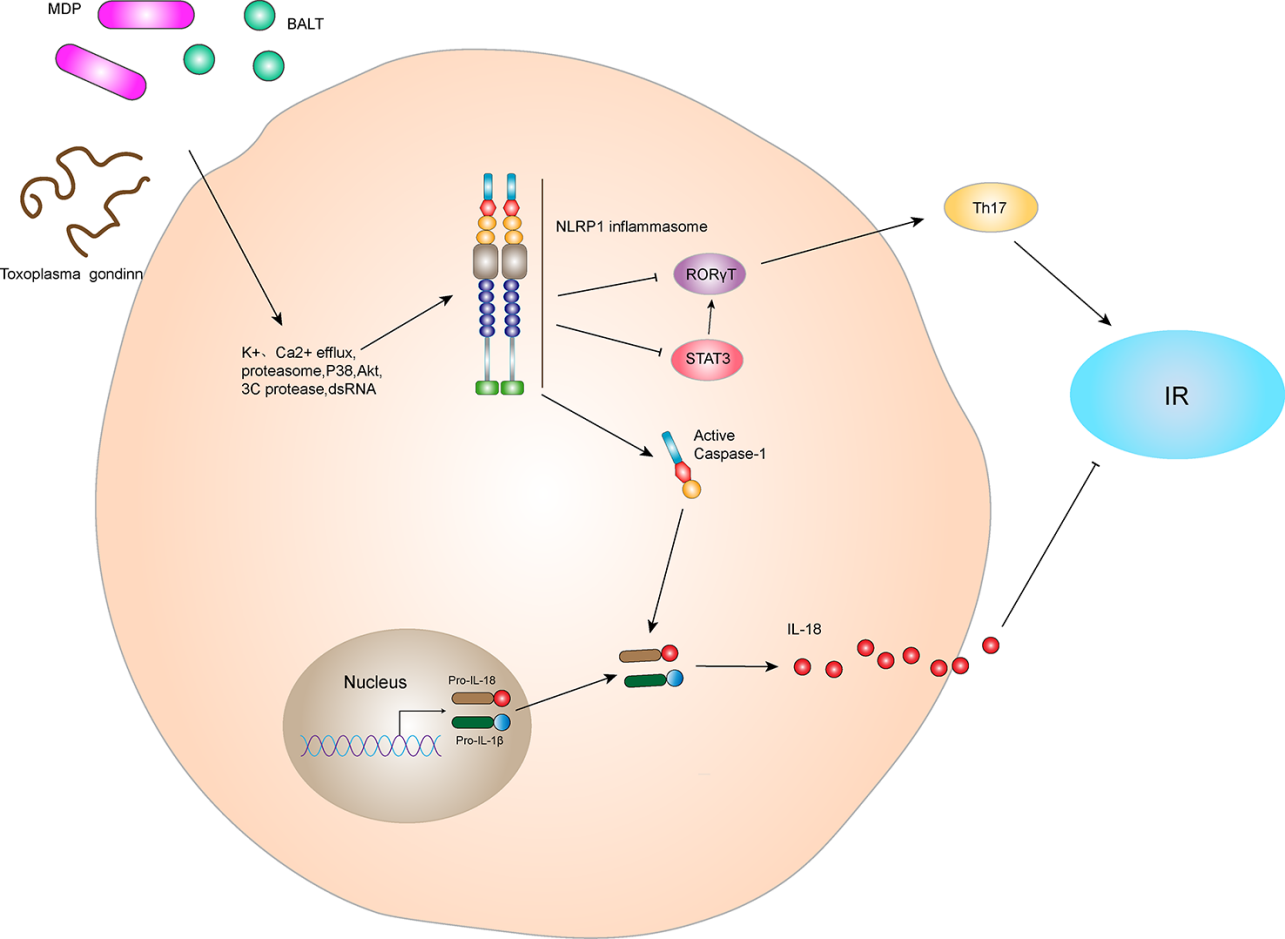
NLRP1 inflammasome and IR. The NLRP1 receptor protein has three active ligands, BALT, MDP, and *Toxoplasma gondii*. After receiving ligand stimulation, NLRP1 receptor proteins can undergo a series of intracellular changes, such as K^+^ efflux, Ca^2+^ efflux, proteasome activation, P38 and Akt inactivation, 3C protease cleavage, and dsDNA stimulation, thereby activating NLRP1 inflammasome. The NLRP1 inflammasome can lead to self-cleavage of pro-Caspase-1 to generate the active form, Caspase-1, which subsequently cleaves pro-IL-18 and pro-IL-1β to generate their mature forms, IL-18 and IL-1β, for secretion. In hyperlipidemia-induced metabolic syndrome, IL-18 inhibits lipid formation and reduces IR. In addition, the NLRP1 inflammasome negatively regulated Th17 cell differentiation and ultimately reduced IR by inhibiting RORγT in the STAT3 pathway.

Inflammation is an important factor in the development of IR and obesity-induced T2DM (23). NLRP1 is an innate immune receptor that plays an important role in metabolic stress. Murphy et al. (24) found that IL-18 produced by NLRP1 inflammasome prevents obesity and metabolic syndrome. Compared with wild-type (WT) mice in the control group, the blood glucose and insulin levels of IPGTT were significantly increased at various time points in NLRP1^-/-^ mice after high-fat diet feeding, indicating that the absence of NLRP1 does not damage the secretion function of pancreatic β cells in mice. NLRP1 mainly affected the blood glucose by reducing IL-18 production, inducing obesity and glucose consumption in mice and leading to significant IR. These data indicate that NLRP1 played a protective role in high-fat-induced metabolic syndrome (**Figure 3**). In addition, NLRP1 has a protective effect in type 1 diabetes (25). After 10 days of streptozotocin injection, blood glucose levels in NLRP1^-/-^ mice were higher than that of WT mice, and inflammatory infiltration in pancreatic islets was increased. This might be related to the fact that NLRP1 expressed by T cells suppresses the negative regulation of retinoic acid-related nuclear orphan receptor-γT (RORγT) in a signal transducers and activators of transcription 3 (STAT3)-dependent pathway, negatively regulating the differentiation of T helper 17 (Th17) cells. In a retrospective and prospective cohort study, NLRP1 also has shown a significant protective effect on diabetic nephropathy (DN) (26). However, Li et al. (27) indicated NLRP1-mediated pro-inflammatory cytokines are significantly increased in the retina of a WT diabetic retinopathy mouse model, whereas the expressions of NF-κB, vascular endothelial growth factor (VEGF), IL-1β, and IL-18 in the serum and retina of NLRP1^-/-^ diabetic retinopathy mice were significantly decreased. Thus, NLRP1 knockdown may reduce levels of inflammatory factors. Additionally, a clinical study revealed that thrombin, the thrombin receptor protease-activated receptor 1, and NLRP1 inflammasome are activated in patients with gestational diabetes mellitus, and their activation aggravates vascular endothelium damage (28). However, the specific mechanism for this damage remains unclear. Therefore, NLRP1 inflammasome may play a different role on diabetes under different status and tissues, and the cause of inflammatory cytokines may be affected by other inflammasomes interfering factor.

### 3.2 NLRP3 inflammasome

#### 3.2.1 Structure and activation of the NLRP3 inflammasome

The NLRP3 inflammasome is the most widely studied inflammasome to date (29). NLRP3 consists of 1016 amino acids and is located at the human coding gene 1q44 (11). It widely exists in immune cells, including macrophages, monocytes, dendritic cells, and neutrophils, as well as central microglia (30). The classical NLRP3 inflammasome consists of the NLRP3 receptor protein, ASC and pro-Caspase-1 (31). The NLRP3 receptor protein also consists of a PYD, NACHT domain, and a LRR domain **(** **Figure 2** **)**. ASC consists of PYD and CARD, which bind to pro-Caspase-1 through CARD-CARD interaction. The PYD promotes homotypic interaction between NLRP3 and ASC. The NACHT domain self-oligomerizes and forms the core of the NLRP3 inflammasome during assembly (29). NLRP3 inflammasome recognizes a variety of risk signals *in vivo* and *in vitro* and is involved in inflammatory reactions contributing to pathological processes in various diseases.

Formation of the NLRP3 inflammasome includes such steps as activation, assembly, regulation and mediates the release of inflammatory factors and pyroptosis. During the whole process, NLRP3 acted as “receptor”, and pro-Caspase-1 acted as “effector”. In the resting state, cellular expression of the NLRP3 protein is below the level required to activate the NLRP3 inflammasome. Exogenous stimuli (such as intense ultraviolet light, cholesterol crystals, uric acid crystals, aluminum salts, silicon dioxide crystals, asbestos, etc.), endogenous stimuli (high ATP concentration released after cell injury, high mobility group protein B1 (HMGB1), purine metabolites, perforin, the saturated fatty acid palmitate (PA), serum amyloid A etc.), pathogen-related irritants (lipopolysaccharide (LPS), bacteria, viruses, etc.) increase NLRP3 protein expression (29, 32).

NLRP3 inflammasome can be activated through both classical and non-classical pathways. The classical activation pathway of the NLRP3 inflammasome mainly includes two stages. The first stage is the initiation stage. NLRP3 promotes downstream NF-κB transcription and increases NLRP3, pro-IL-1β and pro-IL-18 by recognizing PAMPs or DAMPs that activate the Toll-like receptors (TLR) signaling pathway. The second stage is the activation stage. During activation oligomerization of NLRP3, ASC, and pro-Caspase-1 are stimulated, triggering formation of the inflammasome. Currently, K^+^ efflux, Na^+^ influx, Cl^-^ reduction, intracellular Ca^2+^ overload, lysosomal damage, reactive oxygen species (ROS), acidosis, mitochondrial DNA (mtDNA) damage, cytotoxic swelling, and protein kinase activation are recognized activation stimuli of NLRP3 (32, 33). Assembly of inflammasome activates hydrolysis of pro-Caspase-1 protein, forming Caspase-1. Active Caspase-1 processes pro-IL-1β and pro-IL-18 into mature and active IL-1β and IL-18, which is released to further expand the inflammatory response.

Activation of the NLRP3 inflammasome releases large amounts of IL-1β and IL-18 to activate inflammatory programmed cell death, termed pyroptosis (10). Recent studies have shown that gasdermin D (GSDMD) in the digestive tract is an important medium for pyroptosis. GSDMD has an N-terminal cell death domain (GSDMDN^Term^), a central short-chain linking region, and a C-terminal self-inhibition domain. Caspase-1 cleaves GSDMD to remove its C-terminus and release it from intramolecular inhibition. GSDMDN^Term^ combines with phosphatidylinositol phosphate and phosphatidylserine in the cell membrane to form a 1-14 nm pore, leading to intrinsic cellular death. GSDMD-dependent pyroptosis also promotes IL-1β and IL-18 release *via* non-classical NLRP3 inflammasome activation (34). In addition, Caspase-1 is cleaved into several proteins involved in the tricarboxylic acid cycle, which can dramatically reduce the production of cellular energy and cause cell edema (35).

The non-classical activation pathway of NLRP3 does not depend on activation of the TLR signaling pathway. It is mediated by mouse Caspase-11 or human Caspase-4 and Caspase-5. Unlike the classical NLRP3 inflammasome, Caspase-4/5/11 cannot cleave pro-IL-1β and pro-IL-18 to make them mature and active but can lead to pyroptosis (36).

#### 3.2.2 NLRP3 inflammasome and IR

IR and chronic low-grade inflammation are typical features of T2DM. The NLRP3 inflammasome play an important role in the pathogenesis of T2DM and IR (37). Glycolipid metabolites and their derivatives are involved in activation of the NLRP3 inflammasome. Traditionally, researchers have considered glucose metabolism disorders, especially hyperglycemia, as key factors in the initiation of chronic inflammation (38, 39). Further studies revealed that chronic inflammation may be closely related to activation and regulation of NLRP3 inflammasome by aberrant glucose metabolism. Glucose can activate NLRP3 inflammasome by recognizing the pathway of ATP/P2X purinergic receptor 4. In addition, glucose can also induce and promote the expression of thioredoxin interaction protein (TXNIP). TXNIP is an important cofactor of NLRP3 inflammasome, thereby enhancing its activation (38). Islet cells derived from mice with chronic hyperglycemia are stimulated by high extracellular concentrations of blood glucose, which activate NLRP3 inflammasome and induce IL-1β production and secretion to promote IR (40, 41). Wen et al. (42) found that the saturated fatty acid palmitate produced IL-1β and IL-18 by activating the NLRP3 inflammasome and interfered with the insulin signaling pathway in NLRP3, Caspase-1, and ASC gene knockout mice. Meanwhile, Stienstra et al. (43) found that Caspase-1 inhibitors significantly enhance insulin sensitivity in obese mice. These results indicate that the NLRP3 inflammasome plays an important role in regulating adipocyte function and IR. In addition, T2DM is often accompanied by excessive secretion of islet amyloid polypeptide (IAPP), and a large number of IAPP aggregate into IAPP amyloid precipitates that are deposited in islet Langerhans cells. Masters et al. (44) found that mouse macrophages destroy lysosomes by engulfing IAPP, and this process activates the NLRP3 inflammasome to produce a series of inflammatory reactions. Similar to type 2 diabetes, studies have shown that fructose can induce insulin resistance in gestational diabetes mice through the NF-κB-NLRP3 pathway (45).

**Figure 4:**
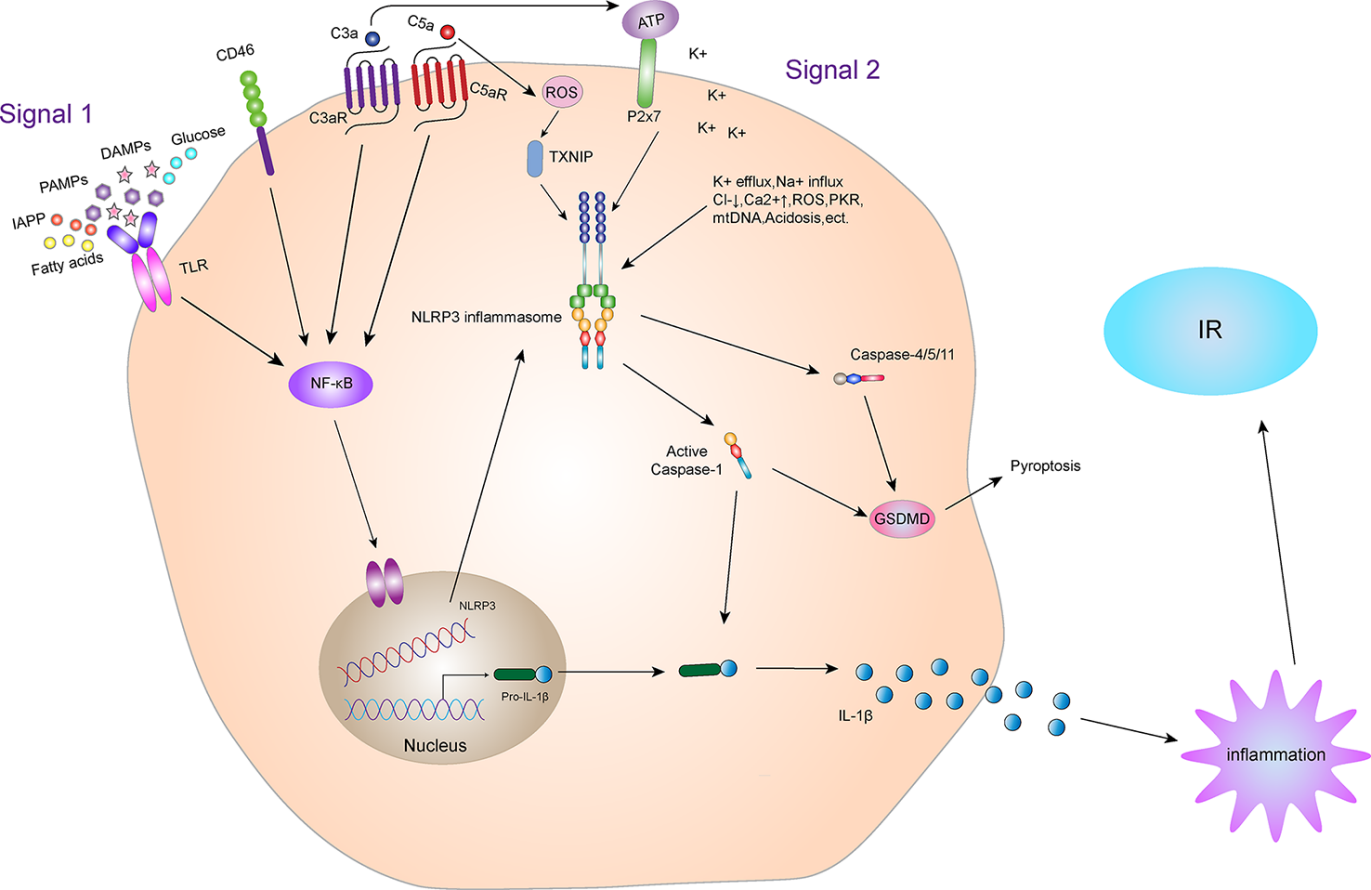
NLRP3 inflammasome and IR. Activation of the NLRP3 inflammasome. Priming and activation of the NLRP3 inflammasome involve two steps. First, inflammasome activation is triggered by priming signals, including various DAMPs and PAMPs, which can lead to NF-κB-mediated upregulation and expression of NLRP3, pro-IL-18, and pro-IL-1β. The second step is inflammasome formation, which is triggered by specific stimuli, the stimuli involved are diverse, including K^+^ efflux, Na^+^ influx, Cl^-^ reduction, intracellular Ca^2+^ overload, lysosomal damage, ROS, acidosis, mtDNA damage, cytotoxic swelling, and protein kinase activation, etc. Complement and its receptors also play a key role in the activation of NLRP3 inflammasome. CD46, C3aR and C5aR can enhance NF-κB to promote the up-regulation and expression of NLRP3, pro-IL-18 and pro-IL-1β. In addition, the C3a-driven ATP efflux leads to increased activation of the ATP receptor P2X7, a potent Signal 2 for NLRP3 inflammasome activation. The C5a can activate the NLRP3 inflammasome by increasing the production of ROS. Activated NLRP3 inflammasome can lead to self-cleavage of pro-Caspase-1 to generate the active form, Caspase-1, which subsequently cleaves pro-IL-1β to generate its mature form, IL-1β, which is secreted out of cells to promote inflammation, leading to IR. Activated Caspase-1 can also induce GSDMD-mediated pyroptosis. In addition, in the non-classical pathway of NLRP3 inflammasome, the NLRP3 inflammasome can also mediate GSDMD-mediated apoptosis through Caspase-4/5/11.

The NLRP3 inflammasome mainly induces IR *via* IL-1β, a downstream signal (**Figure 4**). Absence of IL-1β protects mice from IR induced by a high-fat diet. Youm et al. (46) have shown that IL-1β damages pancreatic islet function, leading to IR. Moreover, after NLRP3 gene knockout, pancreatic β cells are protected due to the decreased secretion of IL-1β. In the obese population, glucose, free fatty acids and ROS-induced endoplasmic reticulum stress led to the activation of JNK signaling pathway, whereas inflammation induced by obesity activates JNK and IKKβ signaling pathways, leading to IR (47). In fact, the activation of NLRP3 inflammasome in adipose tissues, whether in adipocytes or macrophages, is similar to the development of obesity and leads to production of IL-1β, which participates in the formation and development of IR. Therefore, obesity or T2DM models, effective inhibition of NLRP3 inflammasome activation reduces the inflammatory response of β cells, thereby improving insulin sensitivity by inhibiting the production of IL-1β. This outcome may also be achieved through activation of the activating adenosine monophosphate kinase (AMPK)-dependent AMPK/NLRP3/HMGB1 signaling pathway (48).

Moreover, NLRP3 inflammasome and its downstream cytokines, particularly IL-1β, are involved in the development of T1DM. IL-1β induces the migration of proinflammatory cells into pancreatic islets, mediates cytokine-induced beta-cell apoptosis, exerts direct cytotoxic effects on beta-cells, and may be an inflammatory signal in the early stage of T1DM (49, 50). However, the role of NLRP3 in T1DM pathogenesis is not completely understood and further research is required before its clinical application.

### 3.3 NLRC4 inflammasome

#### 3.3.1 Structure and activation of the NLRC4 inflammasome

The NLRC4 inflammasome, also known as the IPAF or CARD12 inflammasome, was first discovered by Poite et al. (51) while studying pre-apoptotic proteins. NLRC4 is located at 2p22.3 in human coding genes and mainly expressed in hematopoietic tissues and cells (11). Like all NLR family members, the NLRC4 receptor protein consists of an N-terminal effector domain CARD, an intermediate-terminal NACHT domain, and a C-terminal LRR **(** **Figure 2** **)**. The NLRC4 inflammasome is composed of the NLRC4 receptor protein, ASC, and pro-Caspase-1. ASC, an adaptor protein, links NLRs at one end and recruits pro-Caspase-1 through the CARD domain at the other end to activate pro-Caspase-1 (52). However, the specific role of ASC in the formation of the NLRC4 inflammasome is still unclear. Some studies believe that formation of the NLRC4 inflammasome requires participation of the adaptor protein ASC, which activates pro-Caspase-1 and eventually causes release of mature IL-1β and IL-18 which participate in the inflammatory response. Some scholars also believe that the NLRC4 receptor protein can directly or indirectly recruit and activate Caspase-1, without participation of ASC (14). However, subsequent studies have revealed that the absence of ASC decreases activation of Caspase-1 and secretion of IL-1β, indicating that ASC plays an important role in the activation of the NLRC4 inflammasome (53).

It is generally believed that activation of the NLRC4 inflammasome is mainly regulated by ligand-binding mechanism, which mainly refers to the bacterial ligand of NLRC4. The NLRC4 inflammasome plays an important role in resisting external bacterial infection. After infected by gram-negative bacteria, such as *Salmonella typhimurium*, *Legionella pneumophila*, *Pseudomonas aeruginosa*, and *Shigella*, assembly of the NLRC4 inflammasome in macrophages plays a key role in activation of Caspase-1 (14). Subsequent studies have found that activation the NLRC4 inflammasome *via* flagellin, the main component in the flagella in all gram-negative bacteria. The flagellin monomer can inject effector proteins into the cytoplasm of host cells through the functional bacteria type III secretion system or type IV secretion system with molecular needle structures that activate the NLRC4 inflammasome (54). To recognize flagellin from different bacteria, the NLRC4 receptor protein must bind to different members of the neuronal apoptosis inhibitory protein (NAIP) family.

When the organism is stimulated by NLRC4-specific ligands, the NLRC4 receptor protein interacts with pro-Caspase-1 and ASC to form the NLRC4 inflammasome (55). Pro-Caspase-1 undergoes dimerization and hydrolysis to active Caspase-1, which in turn cleaves pro-IL-1β and pro-IL-18 into active IL-1β and IL-18 which in turn produce a series of immune responses. In addition, activation of the NLRC4 inflammasome causes lysis of GSDMD, undermining cell membrane integrity and leading to cellular osmotic lysis and cell scorch death. NLRC4 can also interact with other intracellular NLR family members. A study by Wu et al. (56) revealed that when *Listeria monocytogenes* infects macrophages, the NLRC4 inflammasome activates NLRP3 and AIM2 inflammasomes and enhances activation of Caspase-1. Thus, it has been speculated that NLRC4 may be upstream activators of NLRP3 and AIM2 inflammasome and that each inflammatory signal is interrelated.

#### 3.3.2 NLRC4 inflammasome and IR

**Figure 5:**
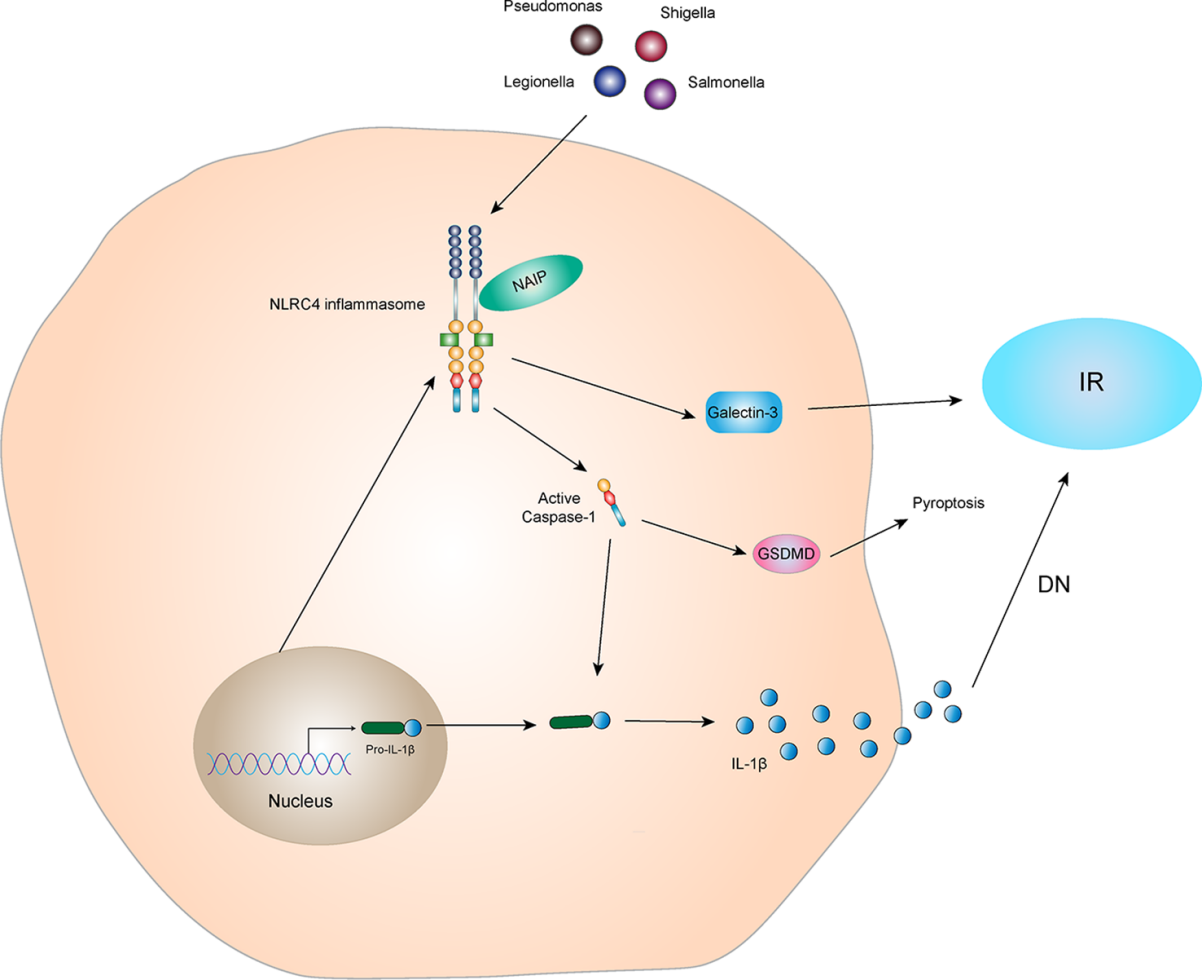
NLRC4 inflammasome and IR The NLRC4 receptor protein can be activated by specific ligands, and the activated NLRC4 receptor protein interacts with pro-Caspase-1 and ASC to form NLRC4 inflammasome. Activated NLRC4 inflammasome can lead to self-cleavage of pro-Caspase-1 to generate the active form, Caspase-1, which subsequently cleaves pro-IL-1β to generate its mature form IL-1β for secretion. Activated NLRC4 inflammasome can indirectly interfere with the conduction of insulin signals and promote the production of IR by promoting the secretion of Galectin-3. IL-1β, which is active in diabetic nephropathy, can mediate kidney tissue damage and IR. In addition, activated Caspase-1 can also mediate the generation of pyroptosis through GSDMD.

Studies have shown that activation of NLRC4 inflammasome leads to an increase in galectin-3 secretion from myeloid cells *via* GSDMD mediation and interferes with insulin signal transduction, leading to IR (57). Diabetic complications are caused by many factors, such as long-term glucolipid metabolism disorders in diabetic patients. IR plays an important role in diabetic complications. Yuan et al. (58) found the expression of NLRC4 is increased in kidney specimens of patients with DN. NLRC4 knockout reduces the inflammatory activity of kidney tissues in mice with T2DM, decreasing excretion of glucose and albumin in the urine and aggregation of macrophages in kidney tissues. These data suggest that the NLRC4 inflammasome aggravates kidney injury by increasing production of IL-1β and aggregation and activation of macrophages (**Figure 5**). In addition, NLRC4, Caspase-1, IL-1β, and IL-18 were significantly increased in the DN mouse model (59). Sequencing results showed that circ_0000181 promoted activation of the NLRC4 inflammasome and release of IL-1β and IL-18, leading to pyroptosis in DN through miR-667-5p/NLRC4 axis regulation. An animal study showed that hyperglycemia upregulated expression of NLRC4 in the gingival tissue of diabetic mice, inducing NLRC4 phosphorylation at Ser533 and activated Caspase-1 (60). Similarly, in macrophages, a high glucose microenvironment induces expression and phosphorylation of NLRC4 and activates Caspase-1, promoting IL-1β production. These results indicate that the NLRC4 inflammasome has a negative regulatory effect in T2DM complications. It is also possible that the NLRC4 inflammasome regulates insulin sensitivity to affect the occurrence and development of T2DM complications.

However, there are few studies on NLRC4 and T1DM or other types of diabetes. A study by Xu et al. (61) in the Han population in China showed that NLRC4 had no significant correlation with T1DM susceptibility. Perhaps NLRC4 does not have a correlation with other types of diabetes, and perhaps more experiments are still needed to explore.

### 3.4 NLRP6 inflammasome

#### 3.4.1 Structure and activation of the NLRP6 inflammasome

The NLRP6 inflammasome, also known as PYPAF5, was first proposed by Grenier et al. (62) in 2002 and is located at 11p15 in the human coding gene (11). NLRP6 is expressed in epithelial cells of various organs, and is also abundant in human peripheral blood mononuclear cells (63). Similar to other NLRP family members, NLRP6 consists of an N-terminal effector domain, PYD, an intermediate-terminal NACHT domain, and a C-terminal LRR **(** **Figure 2** **)**. The amino acid sequence similarities between NLRP6 and NLRP3 are 32% in humans and 33% in mice. The main sequence variation is derived from the LRR domain at the C-terminus, indicating that NLRP6 and NLRP3 have different ligand recognitions (64). A recent cryo-electron microscopic study revealed the structural mechanism of assembly of the NLRP6 inflammasome, where the PYD formed a filamentous structure *via* self-assembly and then underwent a conformational change that enabled NLRP6 to recruit ASC through PYD-PYD homologue protein interaction (65). ASC can form a complete NLRP6 inflammasome by binding to Caspase-1 or Caspase-11 *via* CARD (9, 66, 67). The NLRP6 inflammasome not only mediates secretion and maturation of the pro-inflammatory cytokines IL-1β and IL-18, but also cleaves GSDMD and induces pyroptosis. Additionally, NLRP6 can also function independently of the inflammasome (66, 68).

An increasing number of studies have shown that the assembly and regulation of NLRP6 inflammasome are affected by a variety of microbial components and metabolites. The bile acid derivative taurine induces activation of the NLRP6 inflammasome and promotes secretion and maturation of IL-18, which in turn regulates production of antimicrobial peptides to form a chemical barrier in the intestinal tract that protects it from inflammation (38). In contrast, spermine and histamine may have an inhibitory effect on the NLRP6 inflammasome (69). In addition to microbial metabolites, lipoteichoic acid (70) and LPS (71) bind to and activate NLRP6 inflammasome. Mukherjee et al. (72) found that the deubiquitinating enzyme Cyld inhibits the binding of NLRP6 to ASC, reducing secretion and maturation of IL-18 and preventing excessive inflammatory reactions. NLRP6 expression is also regulated by hormones. In irritable bowel syndrome, increased levels of corticotrophin releasing hormone down-regulate expression of NLRP6 (73, 74).

#### 3.4.2 NLRP6 inflammasome and IR

The NLRP6 inflammasome has different effects in different tissues or diseases, and which has both positive and negative regulatory effects (75, 76). Due to the high expression of NLRP6 in the intestine, current research on NLRP6 function is mainly focused on the intestine (77). Studies have also been conducted in the liver and the lungs, but the relationship between NLRP6 and IR in these tissues is currently unclear. One study has shown a clue between NLRP6 and insulin sensitivity. Rosiglitazone, a drug used to treat with T2DM, can regulate the expression of NLRP6. In this transcriptional expression study, NLRP6 gene is regulated by a variety of transcription factors, including peroxisome proliferator-activated receptor (PPAR) γ, retinoid X receptor (RXR)-α, and chicken ovalbumin upstream promoter transcription factor 1 binding sequences (78). As a member of the nuclear hormone receptor superfamily, PPAR-γ forms a heterodimer with RXR-α and plays a key role in glucose homeostasis (79, 80). Rosiglitazone, as a PPAR-γ agonist, is also a commonly used insulin sensitizer in clinical practice. Studies have shown that stimulation of the human intestinal epithelial cell line Caco-2 with a moderate dose of rosiglitazone for 6 hours significantly increases expression of NLRP6, and there is a dose-dependent relationship between increases in NLRP6 mRNA levels (78).

Moreover, NLRP6 differs from other NLRs family proteins as it is related to inhibition of the innate immune response. It not only participates in the inflammatory response by producing IL-1β and IL-18 but also inhibits NF-κB and MAPK signaling pathways, thereby inhibiting inflammation and preventing pathological damage (81). Studies have shown that expressions of cytokines, such as TNF-α and IL-6, in NLRP6 gene knockout mice increase after macrophages are infected with *Listeria monocytogenes* or are exposed to TLR2/4 ligands, indicating that NLRP6 specifically inhibits activation of TLR2/4-dependent NF-κB and MAPK pathways (82). In alcoholic hepatitis and non-alcoholic steatohepatitis models, NLRP6 inhibits activation of the NF-κB signaling pathway and plays a role in protecting the liver (75, 83). It is predicted that NLRP6 may regulate the insulin sensitivity through the modulatory of inflammatory signals.

**Figure 6:**
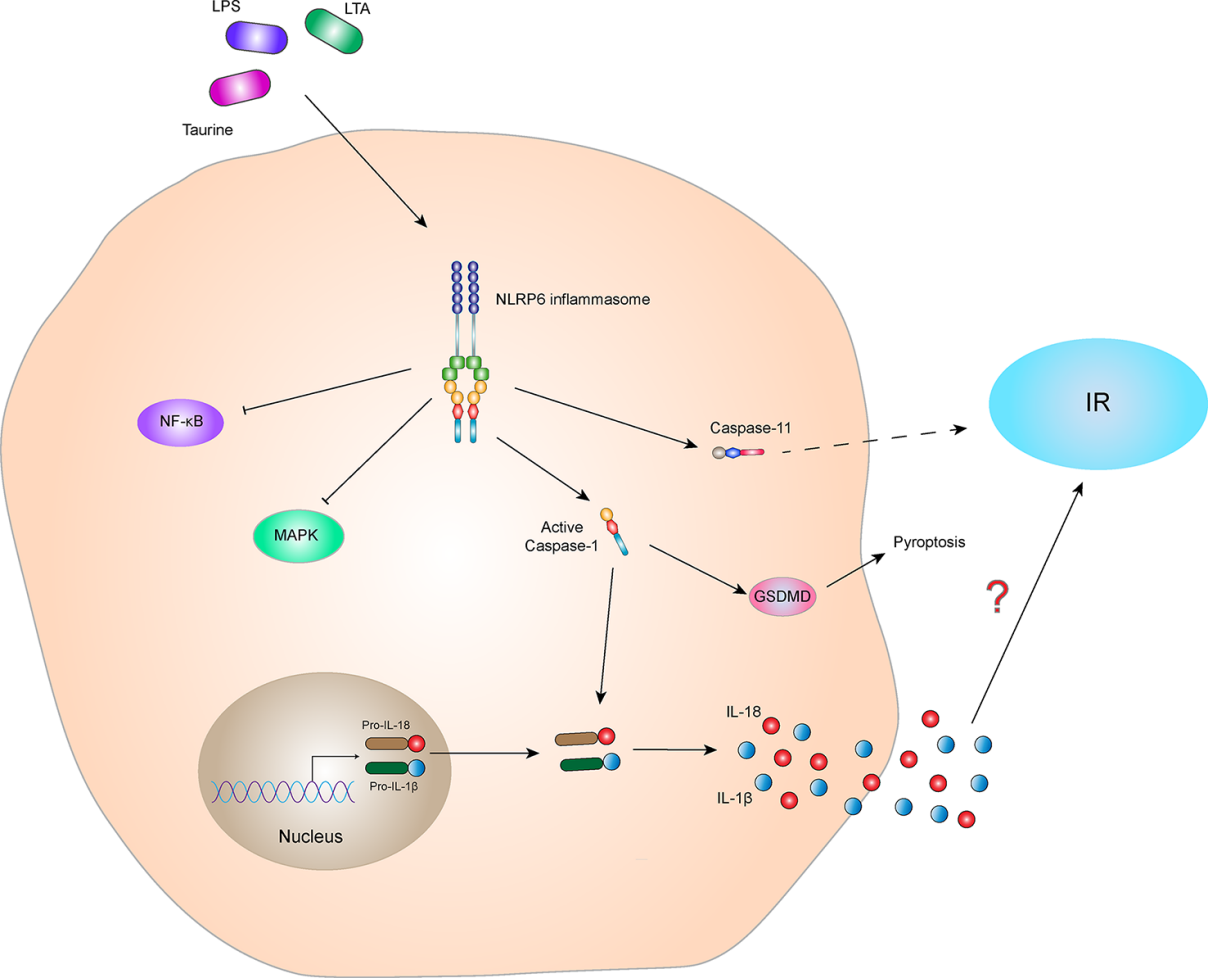
NLRP6 inflammasome and IR. The NLRP6 inflammasome can be activated by the specific ligands, Taurine, LAT, and LPS. The activated NLRP6 inflammasome can lead to self-cleavage of pro-Caspase-1 to generate the active form, Caspase-1, which subsequently cleaves pro-IL-18 and pro-IL-1β to generate their mature forms, IL-18 and IL-1β, for secretion. Activated Caspase-1 can also induce GSDMD-mediated pyroptosis. Activated NLRP6 inflammasome inhibits the NF-κB and MAPK signaling pathways. In addition, activated NLRP6 inflammasome could also activate Caspase-11, indirectly leading to the occurrence of IR. However, the relationship between NLRP6 inflammasome-mediated cytokines (IL-1β and IL-18) and IR is still not clear.

Furthermore, some studies have shown that the NLRP6 inflammasome alters intestinal barrier function by affecting the intestinal mucus layer, intestinal antimicrobial peptides, and colonic mucosal repair. Impaired barrier function may result in bacterial LPS entering the blood, causing metabolic endotoxemia, low-grade inflammation, and metabolic syndromes, such as obesity and IR (84, 85). These results indicate that the NLRP6 inflammasome indirectly affects IR. In addition, studies have shown that the NLRP6 inflammasome activates Caspase-11 and induces loss of gut-associated neurons. After the microbiota is depleted, specific intestinal neurons involved in glucose regulation are absent, resulting in elevated blood glucose and IR (86, 87). Additionally, activation of the NLRP6 inflammasome-induced the secretion of IL-1β and IL-18 probably involved in the regulation of IR (**Figure 6**). In conclusion, there is little research on the correlation between NLRP6 and IR, and the relationship between NLRP6 and IR is not clear. Further study is needed to clarify this relationship and is expected to provide new ideas for the treatment of patients with IR-related metabolic syndrome.

### 3.5 AIM2 inflammasome

#### 3.5.1 Structure and activation of the AIM2 inflammasome

AIM2 was found in melanoma in 1997 (88). Due to its lack of expression in melanoma, it is designated as absent in melanoma 2 (AIM2), belonging to a family of HIN-200 proteins. The AIM2 protein is located at 1q22 in human coding genes. The RNA is 1,485bp in length and consists of 344 amino acid molecules. The molecular weight of the protein is about 39,487 Da, and it is mainly expressed in the cytoplasm (89). The AIM2 protein is highly conserved in the HIN-200 family and usually consists of two parts, the N-terminal PYD and a HIN domain at the C-terminus **(** **Figure 2** **)**. AIM2 inflammasome is composed of AIM2, ASC, and pro-Caspase-1. Under normal physiological conditions, binding of the HIN domain of the AIM2 protein to PYD is in a self-inhibitory state (90). When stimulated by double-stranded DNA (dsDNA), AIM2 recruits pro-Caspase-1 *via* the adaptor protein ASC, forming a complete AIM2 inflammasome and activating Caspase-1. The AIM2 inflammasome then cleaves pro-IL-1β and pro-IL-18 and promotes maturation and release of IL-1β and IL-18. In addition, active Caspase-1 cleaves GSDMD and mediates pyroptosis.

The main function of AIM2 is to identify abnormal cytoplasmic dsDNA in bacteria, viruses, and host cells (91). Since AIM2 does not distinguish between the dsDNA of hosts and microorganisms, it is generally believed that activation of the AIM2 inflammasome is involved in the pathogenesis of many autoimmune inflammatory diseases (92). Activation of the AIM2 inflammasome is not related to the dsDNA sequence but depends on the dsDNA length. Studies have shown that at least 70bp of dsDNA is required to activate the AIM2 inflammasome (93). In addition, people have also found that the longer the dsDNA AIM2, the faster the assembly of the AIM2 inflammasome and that the polymerization rate of the AIM2 inflammasome with dsDNA of 300bp is significantly faster than that with dsDNA of 72bp (94).

Activation of the AIM2 inflammasome is protective during bacterial infection, which requires two basic requirements, bacterial entry into the cytoplasm and release of bacterial DNA by lytic action. AIM2 provides the host with immune monitoring of some pathogenic bacteria, such as *Francisella tularensis*, *Listeria monocytogenes*, *Streptococcus pneumoniae*, *Mycobacterium*, *Porphyromonas gingivalis*, *Staphylococcus aureus*, *Brucella abortus*, *chlamydia murine*, and *Legionella pneumophila* (95). In addition, a variety of DNA viruses can also activate the AIM2 inflammasome, including the *vaccinia virus*, *mouse cytomegalovirus*, and *herpes simplex virus*.

#### 3.5.2 AIM2 inflammasome and IR

**Figure 7:**
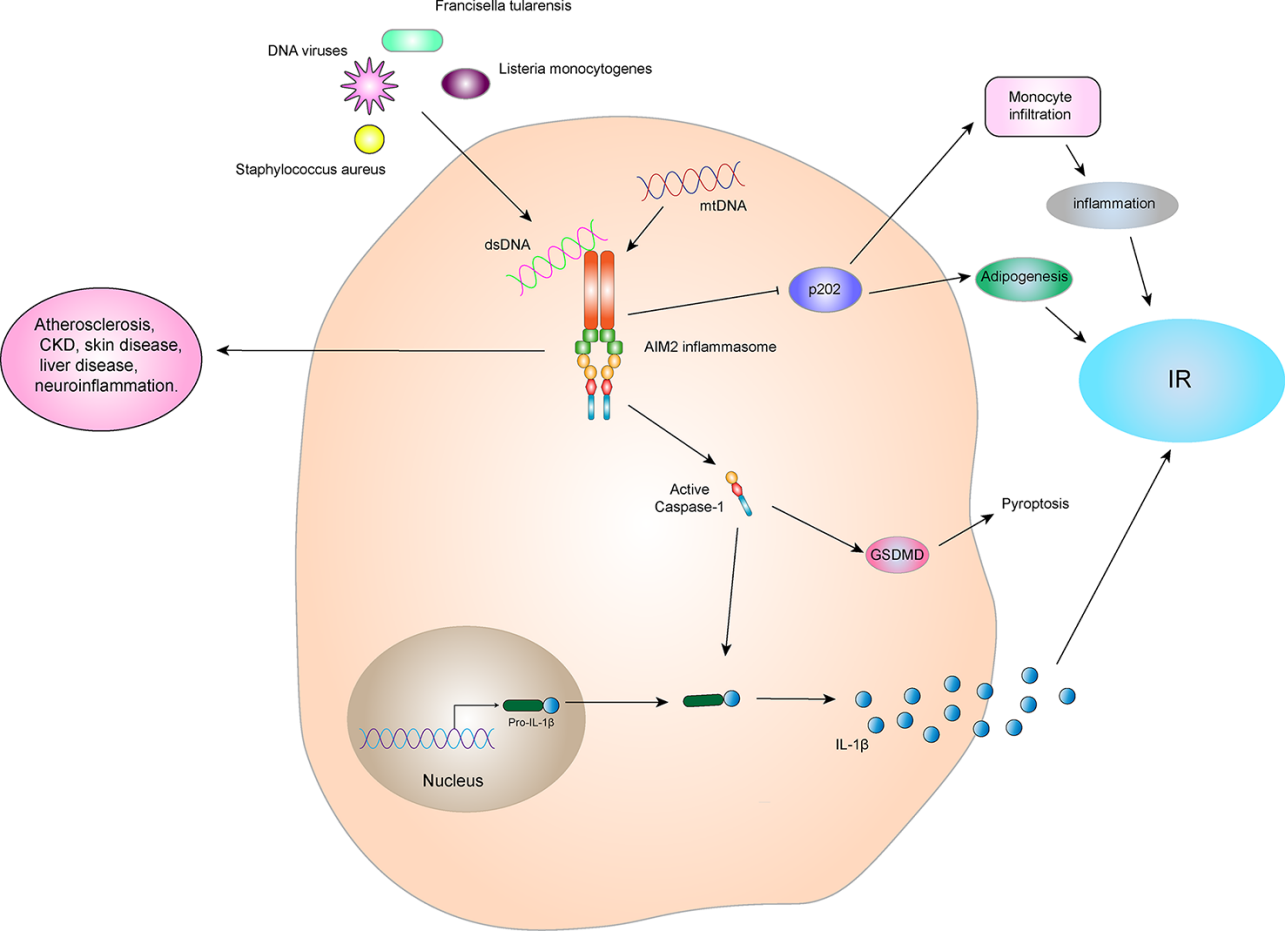
AIM2 inflammasome and IR. The AIM2 inflammasome is activated by dsDNA in the cytoplasm, which then leads to self-cleavage of pro-Caspase-1 to generate the active form, Caspase-1, which subsequently cleaves pro-IL-1β to generate its mature form, IL-1β, for secretion. IL-1β can promote the development of IR and T2DM. Activated Caspase-1 can induce GSDMD-mediated pyroptosis. However, the AIM2 inflammasome can inhibit p202 protein, thereby blocking p202 protein-induced monocyte infiltration and lipogenesis, and indirectly improving obesity and IR. Therefore, the relationship between AIM2 inflammasome and IR depends on the interact protein and downstream signaling.

Although AIM2 inflammasome has a protective effect in infectious diseases, they are harmful in some aseptic inflammatory diseases, including atherosclerosis (96), chronic kidney disease (CKD) (97), skin disease (98), liver disease (99), neuroinflammation (100), and others. The initial progress has been made between the AIM2 inflammasome and IR. AIM2 expression is increased in the monocytes of T2DM patients compared to healthy controls. Similarly, serum cellular mtDNA levels in patients with T2DM are higher than those in the healthy control group and are positively correlated with pathology. Increased mtDNA content in peripheral blood or leukocytes is associated with T2DM nephropathy or hyperglycemia, respectively (101). The expression and activation of AIM2 and the increase in circulating mtDNA levels may be involved in the inflammatory process in patients with T2DM (102). Mitochondrial dysfunction is closely related to IR and the pathogenesis of T2DM (103). Damaged mitochondria release a large amount of mtDNA which participates in activation of the AIM2 inflammasome, increase the expression of IL-1β, and promotes IR and T2DM development **(** **Figure 7**).

In addition, some studies have shown that AIM2 plays an important role in myocardial cell death and fibrosis through the GSDMD pathway in high glucose-induced ROS-mediated diabetic cardiomyopathy, and inhibition of AIM2 expression reduces progression of diabetic cardiomyopathy (DCM) (104). The high glucose environment generated by diabetes can also induce dysfgunction of macrophages through cytoplasmic dsDNA/AIM2-related apoptosis, accelerating the aging process and inducing a systemic pro-inflammatory state (105). By contrast, in an animal experiment, AIM2^-/-^ mice were more prone to obesity, impaired brown fat function, increased fasting blood glucose and insulin levels, and impaired metabolic functions such as IR when compared to WT mice (106). These observations may be due to AIM2 inhibition of the encoded protein 202 (p202), which in turn blocks p202-induced monocyte infiltration and lipogenesis and improves obesity and IR **(** **Figure 7** **)**. This indicates that AIM2 has a protective effect in metabolic syndromes, which is independent on the formation of the inflammasome.

At present, there are few studies on AIM2 and T1DM or other types of diabetes, and AIM2 has been found to play a protective role in the STZ-induced T1DM model in an animal experiment (107). However, more studies are needed to confirm the association of AIM2 with IR and other types of diabetes.

### 3.6 Inflammasome regulation by complement activation

The complement system is a potent component of the innate immune response, promoting inflammation and orchestrating defense against pathogens. Complement activation culminates in a massive amplification of the immune response leading to increased cell lysis, phagocytosis, and inflammation (108). The activation of complement mainly includes three pathways three pathways: 1) the classical pathway with C1q as its main pattern recognition sensor, 2) the lectin pathway with mannose binding lectin and ficolins as the recognition molecules, and finally 3) the alternative pathway with a thioester moiety of C3b as well as properdin as the recognition molecules. Once activated all three pathways converge at C3 (109). Recently, the activation of complement receptors, such as the anaphylatoxin C3a and C5a receptors and the complement regulator CD46, in conjunction with the sensing of cell metabolic changes, for instance increased amino acid influx and glycolysis, have emerged as additional critical activators of the inflammasome. Given that the complement system is evolutionarily among the oldest PRR systems, and that extensive crosstalk between complement and particularly the TLRs exists (110, 111), the involvement of complement in inflammasome activation is not unexpected.

Indeed, studies in the 1980s by Haeffner-Cavallion et al. showed that the anaphylatoxin C3a induces IL-1β production in human monocytes (112), indicating a functional connection between these two systems at a time before the inflammasome was actually discovered. A more recent study defined the C3aR-driven signals in human monocytes and demonstrated that locally produced C3a increases ATP efflux from the monocyte cytosol in the presence of TLR4 activation by LPS (113). This C3a-driven ATP efflux leads to subsequent increased activation of the ATP receptor purinergic ligand-gated ion channel 7 (P2X7), a potent Signal 2 for NLRP3 inflammasome activation (114), and substantially increased IL-1β secretion (113). Furthermore, the anaphylatoxin C5a has been confirmed as an important driver of Signal 1 for NLRP3 inflammasome activation in human monocytes. Samstad et al. (115) showed that cholesterol crystals activate both the classical (via C1q) and alternative complement pathways and that C5a generated during this process, together with TNF-α, functions as priming Signal 1 for NLRP3 activation by increasing IL-1β gene transcription. There is also indication that CD46 partakes in NLRP3 inflammasome priming, as CD46 engagement during T cell receptor stimulation on human CD4^+^T cells potentiates NF-κB activation and increases transcription of IL-1β (116). Studies have shown that C5a (and possibly C3a) can also activate the NLRP3 inflammasome by increasing the production of ROS (117). Conversely, reduced NLRP3 inflammasome activity was also demonstrated in some mouse models with complement and complement receptor-related gene knockout. For instance, Fatemeh Fattah et al. (118) found that the complement protein C6^-/-^ significantly reduced NLRP3 inflammasome levels and pro-inflammatory products (cytokines, chemokines, and histones) in mouse models of polymicrobial sepsis and acute lung injury. Yu et al. (119) found that C5aR2 promotes NLRP3 activation by amplifying dsRNA-dependent protein kinase R expression, which is an important NLRP3-activating factor. In C5aR2^-/-^ mice, the activation of NLRP3 inflammasome and the release of HMGB1 *in vivo* and *in vitro* were obviously restricted. V-set and immunoglobulin domain-containing 4 (VSIG4) is a complement receptor of the immunoglobulin superfamily that specifically expresses in resting tissue-resident macrophages (120, 121). Studies show that VSIG4 inhibits NLRP3 and Il-1β transcription *via* A20-mediated NF-κB inactivation (122).

Complement system plays an important role in the activation of NLRP3 inflammasome. Studies have shown that complement receptor also plays an important role in regulating metabolic syndrome such as T2DM and IR (**Figure 4**). Mamane et al. (123) found that C3a receptor (C3aR) was highly expressed in adipocytes and macrophages in white adipose tissue of mice. In C3aR^-/-^ mice, both ND and HFD showed increased insulin sensitivity, decreased macrophage infiltration and decreased pro-inflammatory factor production. Lim et al. (124) found that in a rat model, obesity, excessive visceral fat, fat inflammation, and insulin resistance caused by the high-fat diet were all related to the increase of plasma C3a, adipose C3aR and C5aR. C3aR and C5aR antagonists can significantly improve the obesity and metabolic disorder in rat model. We can speculate that the deficiency of C3aR and C5aR may be due to limiting the activation of NLRP3 inflammasome, which improves metabolic syndromes such as IR.

### 3.7 Correlation between different inflammasomes

The inflammasomes are innate immune system receptors and sensors that regulate the activation of Caspase-1 and induce inflammation in response to infectious microbes and molecules derived from host proteins. They have been implicated in a host of inflammatory disorders (125). Different inflammasomes play different roles in different stage of that disease and in different tissues or organs. However, there is also a complex correlation between different inflammasomes.

Studies have shown that NLRP1 and NLRP3 inflammasomes exert a synergistic effect and have a protective function on the body in the early stage of type 1 diabetes. However, as the disease progressed, NLRP1 and NLRP3 inflammasomes failed to exert their protective effects during diabetic ketoacidosis and showed significant decline (126). ROS-induced NLRP1 and NLRP3 in monocytes of chronic insomnia disorder also work synergistically to control microglia IL-1β production and the subsequent IL-1β-mediated inflammatory cascade in the brain (127). In addition, Chen et al. found that NLRP12 coordinates with NLRP3 and NLRC4 to induce IL-1β processing and pyroptotic cell death in a manner dependent on the Caspase-8-mediated hypoxia-inducible factor-1α (HIF-1α) pathway during retinal ischemic injury. Furthermore, mature IL-1β initiates the neuroinflammation cascades and pyroptotic reaction by promoting the Caspase-8 mediated HIF-1α-NLRP12/NLRP3/NLRC4 pathway and GSDMD cleavage (128). There was also a synergistic effect of NLRP3 and AIM2 inflammasomes in monocytes from patients with COVID-19, which recognized cell membrane damage and cytosolic DNA, respectively, that activate Caspase-1 and GSDMD, leading to pyroptosis and release of potent inflammatory mediators (129). Studies have shown that NLRP6 and NLRP9b also have synergistic effects in the regulation of *enterovirus* defense. NLRP6 tends to bind to long dsRNA, and NLRP9b binds more strongly to short dsRNA (130, 131). Recent data indicate that NLRP6 is highly expressed in the duodenum (the proximal small intestine), whereas NLRP9b is expressed mainly in the ileum (the distal small intestine), which matches exactly the infection patterns of each virus sensed by these two receptors (64). Thus, NLRP6 and NLRP9b might cooperate in defense against different enteric viruses with different tropisms. The complementary expression pattern, ligand-binding pattern, and downstream signaling they activate make them the perfect pair of NLRs in the intestine.

In addition to synergistic effect, there is also an inhibitory effect among various inflammasomes. Li et al. found that NLRP6 could inhibit p38-MAPK and NF-κB signaling pathways in liver after allogeneic hematopoietic stem cell transplantation and NLRP6^-/-^ could increase the expression of NF-κB, which then activated NLRP3 expression and aggravated liver injury (132). In addition, studies have shown a negative correlation between the expression of NLRP3 and NLRP6 in normal conjunctiva and pterygium. Activation of NLRP3 inhibits the activation of NLRP6 in normal conjunctival and pterygium tissues (133). Studies have shown that during *P. brasiliensis* infection, NLRC4 inhibits Prostaglandin E2 production, which is required for NLRP3-inflammasome-triggered IL-1β at the early phase of infection, while in the later phase of infection reduces IL-18 levels, abrogates CD8^+^IFN-γ^+^T cell responses, and promotes uncontrolled fungal growth (134). It indicated that NLRC4 indirectly inhibited the expression of NLRP3. An animal experiment showed that NLRC4 negatively regulates immunological memory by preventing delayed activation of the cytosolic NLRP3 that would otherwise amplify the production of cytokines important for the generation of Th1 immunity such as IL-18 (135).

The decrease of intracellular K^+^ is a common minimal cellular step for most of the NLRP3 stimuli (136, 137). The linker between the N-terminal PYD, the central NACHT domain and the FISNA domain (a NLRP3-specific domain) are important for activation of NLRP3 when intracellular K^+^ is reduced and K^+^ independent activators are used. However, the NLRP6 is not activated by K^+^ efflux (70, 81). Studies have found that the introduction of the NLRP3 PYD-linker-FISNA sequence into NLRP6 resulted in a chimeric receptor able to be activated by K^+^ efflux-specific NLRP3 activators and promoted an *in vivo* inflammatory response to uric acid crystals. It has been shown that the amino-terminal sequence between PYD and NACHT domain of NLRP3 is a key for inflammasome activation (138).

## 4 Potential treatment options related to the inflammasome

It is crucial to understand immune regulation mechanisms under homeostasis, which may be the key in preventing metabolic syndrome disease, such as IR and T2DM. It has been shown that targeted on inflammasome-related pathways or molecules effectively treats IR and decreases the occurrence and development of T2DM. So far, the NLRP3 inflammasome is the most widely studied inflammasome and the associated medicines are more developed. Studies have shown that a variety of commonly used clinical drugs (**Table 1**), herbal drugs (**Table 2** **)**, protein and peptide drugs (**Table 3**), and NLRP3 inhibitors (**Table 4**) can effectively act on the NLRP3 inflammasome and related downstream molecules to improve IR, T2DM, non-alcoholic fatty liver disease, and other metabolic diseases (174).

**Table 1:** Clinical drugs.

Clinical Drugs	Type	Inhibition mechanism	Refs.
Metformin	Biguanides	Activation the AMPK pathway inhibits Caspase-1 and IL-1 β	(139, 140)
Dapagliflozin	SGLT-2 inhibitors	Activate the AMPK and mTORC2 signaling pathways	(141)
Empagliflozin	SGLT-2 inhibitors	Activate the AMPK and mTORC2 signaling pathways	(142)
Pioglitazone	Thiazolidinediones	Inhibit AGE/RAG signaling pathway and local ROS release	(143)
Acarbose	α- Glycosidase inhibitors	Inhibit Nox4 oxidase	(144)
Liraglutide	GLP-1 receptor agonists	Inhibition of NLRP3 inflammasome activation	(145)
Saxagliptin	DPP-4 inhibitors	Inhibition of NLRP3 inflammasome activation	(146)
Glibenclamide	Sulfonylureas	Inhibits ATP-sensitive K^+^ channels	(147, 148)
Verapamil	Ca^2+^ channel blockers	Enhance Trx-R activity and inhibit TLR4	(149, 150)
Fenofibrate	PPARα agonists	Regulation of Nrf2 signaling	(151)
Vitamin D	Vitamin	Inhibition of ROS/TXNIP pathway and activation of AMPK pathway	(152, 153)
Vitamin E	Vitamin	Inhibit TRAF6/NF-κB pathway and activate AMPK/autophagy axis	(154)

**Table 2:** Botanical drugs.

Botanical Drugs	Inhibition mechanism	Refs.
TSF	Inhibition of ROS production and TXNIP expression	(155)
PN	NACHT ATPase inhibitor and Caspase-1 inhibitor	(156)
Tilianin	Regulation of the Nrf2/TXNIP/NLRP3 inflammasome pathway	(157)
Resveratrol	By regulating the combined effects of Sirt1 and Sirt6	(158)
BBR	Inhibition of NLRP3 inflammasome activation	(159)
Swertiamarin	Inhibition of NOXs/ROS/NLRP3 signaling pathway	(160)
Salidroside	Inhibition of P2X7 receptor expression and regulation of AMPK/NLRP3 axis	(161, 162)
Ginsenoside	Inhibition of oxidative stress	(163)

**Table 3:** Protein and peptide drugs.

Biological inhibitor	Inhibition mechanism	Refs.
DsbA-L	Interaction with Ero1-L; Promote AMPK phosphorylation	(164, 165)
SIRT2	Deacetylation of NLRP3	(166)
HIF2α	Promotion of H3K27me3 modification and inhibition of CPT1A expression	(167)
CWA	Regulate the expression of TXNIP and the activation of p65	(168)

**Table 4:** NLRP3 inhibitors.

NLRP3 inhibitors	Inhibition mechanism	Refs.
MCC950	Binds Walker B motif; NACHT ATPase inhibitor	(169)
CY-09	Binds Walker A motif; NACHT ATPase inhibitor	(170)
Tranilast	Directly binding to the NACHT domain of NLRP3	(171, 172)
Compound C	ATP-competitive AMPK inhibitor	(173)

### 4.1 Clinical drugs

#### 4.1.1 Metformin

Metformin is a first-line drug for the clinical treatment of T2DM. It causes AMPK activation by inhibiting liver glycogen production, gluconeogenesis and IR, and plays a role in a variety of metabolic diseases (175). Lee et al. (139) found that metformin could inhibit Caspase-1 and IL-1β by activating the AMPK pathway, thus improving insulin sensitivity. In addition, another study showed that metformin improves IR and liver fibrosis by reducing ASC and Caspase-1 transcription levels in a T2DM rat model (140). At present, metformin is widely used in clinical practice. Because of its good hypoglycemic effect, especially on fasting blood glucose, and no hypoglycemia when used alone, it can reduce body weight and has cardiovascular benefits. It is the preferred drug for patients with type 2 diabetes recommended by domestic and foreign guidelines. However, gastrointestinal reactions are more common, and taking it during or after meals can reduce gastrointestinal adverse reactions. Patients who use it for a long time should be appropriately supplemented with vitamin B12. Maximum dose for adults is 2550 mg, which is not recommended for children under 10 years of age.

#### 4.1.2 Dapagliflozin and Empagliflozin

Both Dapagliflozin and Empagliflozin belong to the class of sodium-glucose transporter 2 (SGLT-2) inhibitors, which can be used for the treatment of T2DM (176). SGLT-2 inhibitors inhibit SGLT-2 activity, reduce renal reabsorption of glucose, and increase the excretion of urine glucose to lower blood glucose. Chen et al. (141) found that dapagliflozin reduces progression of diabetic cardiomyopathy and the degree of myocardial fibrosis by AMPK and inhibiting activation of the NLRP3 inflammasome. Empagliflozin inhibits activation of the NLRP3 inflammasome by increasing serum BHB levels and reducing serum insulin, thereby significantly reducing secretion of IL-1β and the incidence and mortality of cardiac adverse events in patients with diabetes and cardiovascular disease (142). SGLT-2 inhibitors, as a new type of medicine for the treatment of T2DM, have the effects of reducing body weight, blood pressure and uric acid due to the low risk of hypoglycemia. At the same time, it has kidney and cardiovascular protection, which has been generally recognized by patients. However, it is still noteworthy that some patients are prone to genitourinary infections after taking these drugs.

#### 4.1.3 Pioglitazone

Pioglitazone is an orally active and selective PPARγ agonist with high affinity for the PPARγ ligand-binding domain. Clinically, it is a thiazolidinedione anti-diabetic drug, which can increase the sensitivity of target tissues to insulin and reduce blood glucose, improve blood lipid profile, improve the activity of fibrinolytic system, improve the function of vascular endothelial cells, and reduce C-reactive protein, and has a protective effect on the cardiovascular system. Studies have shown that a specific dose of pioglitazone inhibits secretion of inflammatory cells by inhibiting the advanced glycation end products (AGEs)/receptor for advanced glycation end products (RAGE) signaling pathway and reducing levels of NF-κB and local ROS release (143). These two mechanisms, decreased ROS and down-regulation of NF-κB weaken activation of NLRP3 inflammasome *in vivo*, thereby improving kidney damage in patients with T2DM and DN. Thiazolidinedione anti-diabetic drugs had good effects on patients with abdominal obesity, NAFLD, and obvious IR, and their use alone did not cause hypoglycemia. Because of its slower effect, patients with cardiac insufficiency may increase the risk of heart failure (cardiac function above Class 3 is prohibited), and older women may increase the risk of fractures, which may cause water and sodium retention, leading to slightly increased body weight (more obvious when used in combination with insulin), etc. Therefore, the drug was not used as the first-line medication, and it was recommended to use when the effects of other drugs were unsatisfactory.

#### 4.1.4 Acarbose

Acarbose is an α-glucosidase inhibitor that not only reduces postprandial hyperglycemia, but also inhibits γ-interferon inducible protein 10, MCP-1, macrophage-derived chemokine (MDC), and TNF-α in LPS-stimulated human mononuclear leukemia cells and downregulates phosphorylation of NF-κB-p65 (177). Recent studies have shown that acarbose can improve the vascular permeability of diabetic patients by inhibiting nicotinamide adenine dinucleotide phosphate oxidase 4 (NOX4) and the NLRP3 inflammasome pathway, which may be a potential mechanism whereby acarbose protects individuals from the cardiovascular complications of diabetes (144). α-glucosidase inhibitors are commonly used drugs in clinic, which do not cause hypoglycemia and have the effect of reducing body weight. They are especially suitable for the population which mainly takes carbohydrate intake in China. It is the first-line drug recommended in the current diagnosis and treatment guidelines for metabolic diseases, and the only drug in China that has obtained the indication of impaired glucose tolerance. However, it has gastrointestinal adverse reactions such as abdominal distension and increased exhaust when being used for the first time, so it is prohibited for patients with chronic gastrointestinal dysfunction, diseases possibly worsened by intestinal distension and severe renal impairment, and should not be used for patients under 18 years old and pregnant women.

#### 4.1.5 Liraglutide

Liraglutide, a human glucagon-like peptide -1 (GLP-1) analog, has become a first-line therapy for T2DM (178, 179). GLP-1 receptor agonists promote islet β cell growth, improve IR, reduce lipid deposition, and reduce appetite and body weight (180). Zhu et al. (145) found that liraglutide improves IR and hepatic steatosis by reducing NLRP3 inflammasome expression and IL-1β in the liver. Liraglutide can well control blood glucose and reduce body weight, and is used for treating patients with ineffective blood glucose control caused by metformin and sulfonylurea. There is evidence-based medical evidence that liraglutide has a protective effect on cardiovascular diseases. The common adverse reactions are gastrointestinal discomfort, mainly including nausea, vomiting and diarrhea. However, most patients can gradually adapt to the treatment with the prolongation of the use period.

#### 4.1.6 Saxagliptin

Dipeptidyl peptidase-4 (DPP-4) inhibitor is a commonly used hypoglycemic drug in the clinic and improves levels of endogenous GLP-1 and glucose-dependent insulin-promoting polypeptide. It promotes the release of insulin by pancreatic β cells and inhibits glucagon secretion by pancreatic α cells, thereby improving the insulin level and reducing blood glucose. Saxagliptin is a type of DPP-4 that prevents the occurrence and development of kidney damage in mice with T2DM by reducing activation of the NLRP3 inflammasome and proinflammatory cytokines TNF-α, IL-1β, IL-6, and IL-18 (146). Saxagliptin is generally not hypoglycemic, does not increase body weight, no gastrointestinal reactions, high safety and tolerance. But its general price is high. There are adverse reactions such as headache, dizziness, nasopharyngitis and cough, but the incidence is very low.

#### 4.1.7 Glibenclamide

Glibenclamide is an insulin secretagogue and belongs to sulfonylurea drugs. Its main function is to stimulate β cells to secrete insulin, which acts on ATP-sensitive potassium channel (K_ATP_) on β cell membrane, promoting calcium ion internal flow and increased concentration of intracellular calcium ion, stimulating the emigration of insulin-containing particles and insulin release, and lowering blood glucose. Glibenclamide reduces LPS-induced damage by streptozotocin in diabetic mice by inhibiting activation of NLRP3 inflammasome (147). Dwivedi et al. (148) also demonstrated that glibenclamide inhibits NLRP3 to reduce the levels of glucose, triglycerides, cholesterol, DNA damage, apoptosis, and inflammatory markers in T2DM mice by improving antioxidant status and upregulating the insulin signaling pathway. Sulfonylurea drugs are common hypoglycemic drugs in clinic and have good hypoglycemic effect. It mainly used for patients with T2DM with certain β cell function and patients without sulfonylurea drug taboo. Moreover, it is preferred as a treatment for T2DM patients who are not suitable for metformin or as a combination regimen for other oral hypoglycemic drugs in T2DM patients with poor glycemic control. Sulfonylurea drugs can be combined with any other type of hypoglycemic drugs with different hypoglycemic mechanisms; however, it should be careful not to use two insulin secretagogues at the same time. Its disadvantages are the risk of hypoglycemia and weight gain. In principle, sulfonylureas are prohibited for patients with liver and kidney insufficiency.

#### 4.1.8 Verapamil

Verapamil is a calcium channel blocker that improves IR and hyperglycemia in patients with metabolic syndrome. In fact, many clinical observations in human and animal models provide evidence for the beneficial effects of calcium channel blockers on obesity-related metabolic pathology (181). Verapamil has been widely used for the treatment of hypertension and the control of angina pectoris. Verapamil enhances thioredoxin reductase activity and significantly inhibits TLR4-mediated NLRP3 inflammasome assembly in diabetic retinopathy mice, reducing the release of inflammatory markers (TNF-α and IL-1β) into the vitreous humor and inhibiting pathological angiogenesis to prevent the development of diabetic retinopathy (149). In addition, verapamil improves pre-diabetic neuropathy in high fat diet-induced obese mice by inhibiting upregulation of TXNIP. These results indicate that verapamil not only has good therapeutic effects on hypertension and cardiovascular diseases but may also have wide application in the field of T2DM and its complications (150).

#### 4.1.9 Fenofibrate

Fenofibrate, a PPARα agonist and a triglyceride-lowering drug, has been widely used in clinical practice for more than 30 years. Fenofibrate has extensive and beneficial effects on multiple signaling pathways of oxidative stress, inflammation, angiogenesis, and cell survival, as well as on the development of diabetic complications (182). In an animal experiment, it was found that fenofibrate reduced diabetes-induced retinal leukostasis and vascular leakage in mice and improved diabetic retinopathy by regulating nuclear factor E2-related factor 2 (Nrf2) signaling and inhibiting NLRP3 inflammasome activation (151). However, fenofibrate is not used as a routine treatment for diabetic retinopathy, which may be related to its side effects, leading to elevated transaminases and impaired liver function.

#### 4.1.10 Vitamin D

Vitamin D is a multifunctional hormone because it as an active metabolite, 1,25-dihydroxyvitamin D3, play a classical role in calcium and bone homeostasis. It has many biological functions, including blood pressure control, immune regulation, apoptosis inhibition, and anti-vascular production. In addition, vitamin D3 plays an important role in endothelial function and has antioxidant and anti-inflammatory effects. Vitamin D3 can protect from retinal vascular injury and apoptosis in mice with diabetic retinopathy by inhibiting the ROS/TXNIP/NLRP3 inflammasome pathway (152). Other studies have shown that vitamin D3 can activate AMPK and inhibit the mTOR pathway, thereby inhibiting activation of the NLRP3 inflammasome and reducing damage of pancreatic β cells (153). Vitamin D3 can promote the reabsorption of calcium and phosphate by renal tubular cells, increase the concentration of blood calcium and phosphorus, and facilitate the generation of new bones and calcification. It is currently used for the prevention of rickets and has not been routinely used for the treatment of diabetes. However, vitamin D3 may have potential value in the treatment of diabetes and its complications.

#### 4.1.11 Vitamin E

Gamma-tocotrienol (γT3), an isomer of unsaturated vitamin E, is known to exert potent anti-inflammatory effects in a variety of cells, including macrophages, adipocytes, and several cancer cells (183). Kim et al. (154) found that γT3 regulates NLRP3 inflammasome through a dual mechanism. The induction of A20/TNF-α induced protein 3 inhibits the TNF receptor associated factor 6 (TRAF6)/NF-κB pathway and activation of the AMPK/autophagy axis results in weakening of Caspase-1 cleavage. Therefore, the infiltration of immune cells into adipose tissue is decreased, circulating IL-18 levels are reduced, pancreatic beta cells are protected, insulin sensitivity is improved, and the progression of T2DM is delayed. However, more favorable evidence is needed to prove that γT3 can be used for T2DM treatment.

### 4.2 Botanical Drugs

#### 4.2.1 Tangshen formula

TSF is a prescription composed of 7 Chinese herbal medicines including 35.3% astragalus root (*Astragaliradix*), 17.6% burning bush twig (*Euonymi ramulus*), 14.4% rehmannia root (*Rehmanniae radix*), 11.5% bitter orange (*Aurantii fructus*), 10.6% cornus fruit (*Corni fructus*), 7.1% rhubarb root and rhizome (*Rhei radix et rhizoma*), and 3.5% notoginseng root (*Notoginseng radix*). It is used to treat diabetic nephropathy and provide renal protection. Studies have shown that TSF reduces production of ROS and the expression of TXNIP, playing an anti-pyroptosis role *via* the TXNIP-NLRP3-GSDMD axis and reducing the degree of renal damage in DN (155).

#### 4.2.2 Parthenolide

PN is a sesquiterpene lactone, which shows anti-inflammatory activity by inhibiting the activation of NF-κB. It can also inhibit histone deacetylase 1 (HDAC-1) protein without affecting other class I/II HDACs (184). As a key component of plant drugs, PN has been widely used to treat a variety of inflammation-related diseases. PN inhibits activation of Caspase-1 by catalyzing alkylation of Caspase-1 cysteine residues or directly targeting the ATP enzyme activity of NLRP3 *via* cysteine modification. Kumar et al. (156) also found that PN reduces obesity-induced inflammation and IR by directly inhibiting NLRP3 inflammasome. PN can be used as a potential therapeutic agent for reducing obesity-induced IR caused.

#### 4.2.3 Tilianin

Tilianin is an active flavonoid glycoside, which is widely distributed in a variety of medicinal plants. It lowers blood pressure, protects the myocardium, reduces blood lipids, and has anti-diabetic, anti-inflammatory, and anti-oxidative effects. Tilianin partially lowers blood glucose, oxidative damage, and inflammation by regulating the Nrf2/TXNIP/NLRP3 inflammasome pathway in mice with diabetic retinopathy and reduces the occurrence and development of diabetic retinopathy (157).

#### 4.2.4 Resveratrol

Resveratrol, a polyphenol found in grapes, mulberries, and red wine, has antioxidant, anti-inflammatory, heart-protecting, and anticancer properties. It has a wide range of targets, such as mTOR and JAK. Resveratrol is not only a specific silent information regulator factor 2-related enzyme 1 (Sirt1) activator, but also an Nrf2 activator, which can improve the aging-related progressive kidney injury in the mouse model, but also act on the endothelial cells to promote NO production. Human clinical trials in diabetic subjects have shown that resveratrol prevents and improves IR and diabetes (185). It has been suggested that these beneficial effects of resveratrol are partially mediated by its antioxidant and anti-inflammatory properties (186). However, its specific mechanism of action remains unclear. Studies have shown that resveratrol may inhibit the activation of NLRP3 inflammasome *via* the combination of Sirt1 and Sirt6, thus exerting a series of protective effects on the body (158).

#### 4.2.5 Berberine

BBR, an isoquinoline alkaloid extracted from Chinese herbal medicine, is one of the main components of *Coptis chinensis* and is used in treating diabetes and dyslipidemia. Studies have shown that berberine can induce ROS production and inhibit DNA topoisomerase, with anti-inflammatory and anti-tumor properties (187). Zhuo et al. (188) found that berberine improves obesity-induced inflammation and IR by triggering macrophage autophagy in order to inhibit PA-induced activation of NLRP3 inflammasome. A recent study revealed that BBR improves high glucose-induced tubular epithelial-mesenchymal transition and renal interstitial fibrosis in DN by inhibiting NLRP3 inflammasome (159). These results indicate that BBR might be a new drug for the treatment of obesity and diabetic complications.

#### 4.2.6 Swertiamarin

Swertiamarin is the active ingredient in many traditional Chinese medicines. Studies have shown that swertiamarin and its derivatives have anti-diabetes and anti-hyperlipidemia effects and can alleviate pathological neuropathic pain (189). However, its anti-diabetic mechanism is still unclear. Swertiamarin corrects the imbalance of diabetic peripheral neuropathic pain (DPN) inflammatory factors and improves DPN by inhibiting the NOXs/ROS/NLRP3 signaling pathway in a rat model of DPN (160).

#### 4.2.7 Salidroside

Salidroside, a phenylpropanoid glycoside compound, is the active ingredient of Radix Rhodiolae. It exerts a beneficial effect in alloxan-induced diabetic mice through antioxidation. In an animal experiment, salidroside inhibited activation of the NLRP3 inflammasome by inhibiting the expression of P2X7 receptor, thus improving pathological pain in diabetic rats (190). Zheng et al. found that salidroside improves IR and diabetic neuropathic pain in diabetic mice by regulating the AMPK-NLRP3 inflammasome axis (161, 162).

#### 4.2.8 Ginsenoside

Ginsenoside is one of the main active components of the precious Chinese medicinal *Radix Ginseng* and has anti-oxidative stress, anti-inflammatory, anti-aging, neuroprotective effects. It has been found that ginsenoside Rg1 significantly reduces expression of NLRP3, Caspase-1, IL-1β, and VEGF in a T2DM mouse model, reducing pathological retinal damage (191). In addition, ginsenoside Rg5 plays an important role in diabetes. Ginsenoside Rg5, the main component of Red ginseng, inhibits the mRNA expression of cyclooxygenase-2 (COX2) *via* suppression of the DNA binding activities of NF-κB p65 (192). In a DN mouse model, Rg5 reduced the inflammatory response by inhibiting oxidative stress, activating NLRP3 inflammasome, and reducing fasting blood glucose, IR, serum creatinine, etc. in DN mice. It also improved kidney damage in diabetic mice (163).

### 4.3 Protein and peptide drugs

#### 4.3.1 Disulfide bond A oxidoreductase-like protein

DsbA-L is an antioxidant enzyme that inhibits endoplasmic reticulum stress and improves adiponectin secretion by interacting with endoplasmic reticulum chaperone Ero1-Lα (164). Yang et al. found that DsbA-L inhibits activation of the NLRP3 inflammasome by promoting AMPK phosphorylation in a DN mouse model, thereby reducing renal fibrosis and renal tubular damage (165). DsbA-L is expected to provide a new avenue for treating DN.

#### 4.3.2 Silent information regulator 2

Nicotinamide adenine dinucleotide (NAD+)/SIRT2 plays an important role in energy homeostasis and cellular metabolism. He et al. (166) showed that SIRT2 deacetylates NLRP3 and inactivates NLRP3 inflammasome. This interaction prevents and reverses inflammation and IR related to aging. In general, increasing the concentration of NAD+/SIRT2 can treat diseases related to IR.

#### 4.3.3 Hypoxia-inducible factor 2α

HIF2α is activated in a hypoxic microenvironment, such as in the adipose tissue of T2DM patients. Li et al. (167) found that HIF2α in macrophages inhibits expression of carnitine palmitoyl transferase 1A (CPT1A) by promoting modification of lysine 27-trimethylated histone H3 (H3K27me3) and preventing NLRP3 inflammasome activation, thereby reducing IR induced by a high fat diet. These results indicate that inhibiting macrophage HIF-2α activation of NLRP3 inflammasome may be a potential therapeutic target for high fat diet-induced IR and other chronic metabolic inflammatory diseases.

#### 4.3.4 Cathelicidin-WA

CWA is an antimicrobial peptide derived from the genus Mushroom, which has a variety of anti-inflammatory effects. CWA inhibits formation of the NRLP3 inflammasome by regulating expression of TXNIP and activation of p65 in a mouse model of DCM. It reduces myocardial fibrosis, inflammation and oxidative stress, and myocardial cell apoptosis in DCM mice and improves heart dysfunction caused by diabetes (168).

### 4.4 Inhibitor

#### 4.4.1 MCC950

The small molecule MCC950, a diarylsulfonylurea-containing compound, was the first small molecule to be identified as an NLRP3 inhibitor. MCC950 blocks activation of NLRP3 inflammasome and the production of IL-1β by inhibiting oligomerization of ASC. MCC950 can directly interact with the Walker B motif in the NACHT domain of NLRP3 and prevent ATP hydrolysis, inhibiting NLRP3 activation and inflammatory soma formation (169). In the diabetic mouse model, 4 months of continuous treatment with MCC950 decreased plasma insulin levels and increased insulin sensitivity (193). In addition, MCC950 treatment regulates expression of peripheral and central stress signals and inflammation in mice. Animal experiments have shown that treatment with MCC950 improves anxiety and depressive behaviors and cognitive dysfunction in diabetic encephalopathy mice and reduces the expression of NLRP3-related inflammatory components in the hippocampus in a mouse model of DEP. MCC950 also improves insulin sensitivity in mice (194). Diabetic retinopathy is an inflammation-mediated microvascular disease characterized by dysfunction of human retinal endothelial cells (HRECs). Significant upregulation of NLRP3, Caspase-1, and IL-1β occur in CD31+ HRECs obtained from patients with diabetic retinopathy. MCC950 treatment of HRECs stimulated by high glucose reduces apoptosis, inhibits interaction of NEK7 with NLRP3, and enhances the function of HRECs (195). DN is another chronic inflammatory microvascular complication of diabetes. Previous studies have reported the role of NLRP3 inflammasome in the pathogenesis of DN. MCC950 reduces levels of tumor growth factor-β, Caspase-1 and IL-1β in high glucose-treated rat glomerular mesangial cells. In addition, animal models of DN treated with MCC950 show improvement in glomerular basement membrane thickening, renal function, renal fibrosis, and podocyte injury (196). Although not yet clinically applied, MCC950 represents a potential therapeutic agent for the treatment of diabetes and its complications.

#### 4.4.2 CY-09

CY-09 significantly inhibits formation of the NLRP3 inflammasome *in vivo* and *in vitro* in human cells in mouse model and is a direct and effective NLRP3 inhibitor (170). CY-09 directly interacts with the NLRP3 Walker A motif and inhibits ATP binding to NLRP3. CY-09 dose-dependently inhibits Caspase-1 activation and IL-1β release by sodium urate, ATP, and Nigericin-induced NLRP3 stimulation in bone marrow-derived macrophages. In a high fat diet-induced mouse model, CY-09 increased insulin sensitivity (197). In another mouse model, sleeve gastroplasty combined with CY-09 reduced body weight, improved IR and reduced hepatic steatosis (198). These data indicate that CY-09 has very considerable prospects in that treatment of metabolic syndrome.

#### 4.4.3 Tranilast

Tranilast is a tryptophan metabolite analogue. It can inhibit the production of prostaglandin D2 and angiotensin II, has anti-inflammatory and immunomodulatory effects, and is used as an anti-inflammatory agent for the treatment of inflammation-related diseases. Studies have shown that tranilast can directly bind to the NACHT domain of NLRP3 and block NLRP3 oligomerization to inhibit NLRP3 inflammasome assembly (171). Cao et al. (172) revealed that tranilast improves hyperglycemia, insulin deficiency, glucose intolerance, IR, and other symptoms of gestational diabetes by reducing the high expression of NLRP3 inflammasome and pro-inflammatory factors. At present, tranilast is not used routinely in the treatment of diabetes, and more studies are needed to prove its safety and stability.

#### 4.4.4 Compound C

Compound C is a pyrazolopyrimidine that is widely used as a competitive inhibitor of cell permeability to ATP of AMPK. Compound C significantly reduces IR in high fat-induced obese mice by downregulating expression of the NLRP3 inflammatory body components and pro-inflammatory markers (173). Compound C can be used as a therapeutic agent for the treatment of metabolic diseases, such as T2DM and NAFLD, but the current research is insufficient and further research is needed.

### 4.5 Gene editing

As a third-generation gene editing tool, CRISPR/Cas9 can specifically and effectively destroy or repair pathogenic genes with a single guide RNA (gRNA)-directed Cas9 nuclease. Xu et al. (199) utilized CRISPR/Cas9 to directly destroy NLRP3 at the genomic level, not only completely inhibiting the activation of NLRP3 inflammasome but also avoiding the potential risk of inhibiting anti-inflammatory biological agents and the off-target pathways of inhibitors. Subsequently, screening an optimized cationic lipid-assisted nanoparticle (CLAN) to deliver Cas9mRNA (mCas9) and gRNA into macrophages is developed. By using CLAN encapsulating mCas9 and gRNA-targeting NLRP3 (gNLRP3) (CLANm_Cas9/gNLRP3_), disrupting NLRP3 of macrophages, inhibiting the activation of the NLRP3 inflammasome in response to diverse stimuli. CLANm_Cas9/gNLRP3_ treatment improves insulin sensitivity and reduces adipose inflammation of high-fat-diet (HFD)-induced T2DM. CLAN_mCas9/gNLRP3_ provides a new treatment strategy for NLRP3-dependent inflammatory diseases.

## 5 Concluding remarks

T2DM, as the most common metabolic disease in the world, is pathologically characterized by IR, which subsequently leads to glucolipid metabolism disorder. T2DM is affected by lifestyle factors such as age, pregnancy and obesity, and has a strong genetic component (200). In recent years, the number of patients with metabolic syndrome accompanied by IR is increasing. With the deepening of research on T2DM, the concept of T2DM as a chronic inflammatory disease has gradually been widely accepted (41, 201, 202). Although there are many influencing factors for IR, such as abnormal glucolipid metabolism, hypoxia, and endoplasmic reticulum stress, a common pathway is ultimately mediated by the inflammatory response (203, 204).

The enhanced inflammatory trait of the adipose tissue during obesity instigates the production of cytokines that contribute to the development of IR (43). As an important part of the innate immune system, inflammasomes play an important role in IR-induced metabolic diseases. With the serious imbalance of metabolic disorder, increase of ROS and damage of mitochondria caused by chronic inflammation, the activation of NLRP3 inflammasome is activated. A large number of studies have shown that the activation of NRLP3 inflammasome affects the interaction of glucose tolerance, insulin sensitivity and intestinal microorganisms, leading to the occurrence and development of T2DM (205–207). On the contrary, with the development of T2DM, multiple organ dysfunctions occurs, leading to inflammation, further activating the activation of NLRP3 inflammasome. In short, T2DM leads to inflammasome activation or vice versa and there is a vicious circle. With extensive research on the structure, assembly, activation, regulation, and the mechanisms of action of inflammasomes, an in-depth and scientific understanding of the relationship between inflammasomes and IR has been obtained. NLRP3 inflammasome is an excellent target for IR and T2DM treatment. Current strategies against NLRP3 inflammasome mainly include anti-inflammatory biological agents against IL-1β signaling or blocking partial upstream and downstream signals of NLRP3 inflammasome.

In addition, the treatment and intervention for T2DM mainly rely on the maintenance of drugs to control blood glucose, which cannot fundamentally reverse the process of T2DM. In fact, whether it is based on insulin sensitization and the use of first-line treatment drugs for T2DM, such as glitazones and metformin, or GLP-1 analogues and dipeptidyl peptidase inhibitors, which have been approved and marketed by the FDA in recent years, there are different degrees of toxic and side effects and blood sugar control failure in clinical practice. Therefore, it is urgent to explore the pathogenesis and intervention strategies of T2DM from a new perspective. Although NLRP3 inflammasomes are closely related to IR and T2DM, research on the treatment of T2DM targeting NLRP3 inflammasomes is still in its infancy. There are three promising therapeutic agents for T2DM that target IL-1β and Caspase-1: (1) IL-1 receptor antagonist Anakinra (208), (2) human anti-IL-1β monoclonal antibody Canakinumab (209), (3) Caspase-1 inhibitor Pralnacasan. Anakinra and Canakinumab are currently in the Phase II clinical trials and have not been officially widely used in clinical practice. Clinical trials of Pralnacasan have been discontinued due to its safety and tolerability (210). In addition, inhibitors of NLRP3, such as MCC950 and CY09, have achieved good results in animal models, but have not been applied to clinical trials.

Although the more detailed molecular mechanism of activation of NLRP3 inflammasomes remains to be clarified until now, and the research on the treatment of T2DM targeting NLRP3 inflammasomes is filled with a large number of unknown challenges, we still believe that challenges are often accompanied by opportunities. Moreover, there are still many questions that require further exploration, for example, in addition to the NLRP3 inflammasome, other inflammasomes have shown the specific relations with IR and T2DM. Furthermore, different inflammasomes exert different effects on IR in different tissues or cells. Meanwhile, they can also become a therapeutic target. Therefore, in the future, deeper and more comprehensive studies on inflammasomes are needed to provide new methods for the diagnosis and treatment of various IR-related metabolic diseases and new ideas for the targeted design of new drugs or regimens.

## Author contributions

SL was responsible for preparing the original draft; YL, ZQ, TZ and ZF were responsible for providing the material and conducting the investigation; LY and XW were responsible for conceptualization and funding support. All authors contributed to the article and approved the submitted version.
